# Examining motivational profiles in the dark personality tetrad using an approach-avoidance conflict task

**DOI:** 10.1371/journal.pone.0327609

**Published:** 2025-07-29

**Authors:** Luke B. J. Regehr, Douglas A. Williams, Erin E. Buckels

**Affiliations:** University of Winnipeg, Winnipeg, Manitoba, Canada; Private University Schloss Seeburg: Privatuniversitat Schloss Seeburg, AUSTRIA

## Abstract

The present studies evaluated motives associated with the Dark Tetrad traits (Machiavellianism, narcissism, psychopathy, and sadism) using a computerized approach-avoidance conflict task (AACT). Study 1 used an Emoticon AACT with a forced choice between smiley and frowny face icons. In Phase 1, participants (*n* = 197) were shown a positive image if they chose to move a stick-figure manikin toward the smiley icon, and were shown a negative image if they chose the frowny icon. In Phase 2, they were offered a varying number of points (0, 1, 5, 25, or 50) for choosing the frowny icon. We found that sadism, Machiavellianism, and psychopathy were associated with accepting fewer points to approach the frowny icon that cued a negative image. This same pattern manifested for participants with low empathy levels, particularly when affective resonance was low and affective dissonance was high. Study 2 (*n* = 191) used an Image AACT where the choice alternatives were positive and negative images, which produced smiley or frowny icons, respectively. Sadism and dissonant emotional tendencies predicted choices directed toward negative images in Phase 2. In Studies 3 (*n* = 288) and 4 (*n* = 276), we confirmed our Emoticon AACT findings using gender-balanced samples. Studies 3 and 4 also introduced a viewing-time task (VTT). Sadism and dissonant emotional tendencies predicted decreased viewing times for positive images, but did not predict increased viewing times for negative images, suggesting that negative emotional reactions produced by the positive images were the primary motivating factor in the present AACT. Overall, our findings serve as further evidence of the different motives underlying socially aversive tendencies.

## Introduction

Graphic images of people in distress, animal cruelty, and interpersonal violence are stimuli that most people would typically prefer to avoid. Evidence suggests, however, that individuals who exhibit malevolent personality traits may instead prefer to engage with these types of aversive images. For example, individuals with high levels of sadism—a tendency to enjoy cruelty [1]—find violent images to be intrinsically rewarding [2,3]. Conversely, they may feel a mixture of negative and positive emotions when viewing other images most people would label positive, such as images of pleasant and joyful interpersonal interactions or peaceful scenes [2,3]. The more general tendency to feel contrary emotions in the presence of others is called affective dissonance [4]. Both sadism and affective dissonance are associated with aggression and violence [4,5], which highlights the role of aberrant emotional responding in supporting antisocial behaviour. Given their social importance, more research is needed to understand the emotional approach and avoidance patterns involved in these tendencies.

Sadism is one of the traits that comprise the Dark Tetrad of personality, which also includes psychopathy, Machiavellianism, and narcissism. This constellation of socially aversive traits is marked by higher levels of disagreeableness and callousness (i.e., low empathy), alongside behavioural tendencies of self-promotion, exploitation, duplicity, and aggressiveness [6–8]. Much of the recent Dark Tetrad literature is focused on uncovering differences between the four traits. Despite their similarities, the literature suggests that the Dark Tetrad are empirically and conceptually distinct [9,10]. For example, psychopathy is distinguished by a lack of remorse, impulsivity, and stimulation-seeking [11,12], which may lead to antisocial behaviours, such as high-risk cheating [13], heavy episodic drinking [14], vengeance [15], and romantic revenge [16]. Distinguishing features of Machiavellianism are cynicism, interpersonal manipulation, and a drive to succeed at the expense of others [6,17], evidenced by unscrupulous behaviour like plagiarizing essays [18], manipulating relationships through exclusion, gossip, and rumours [19], and using high-risk high-reward strategies in gambling tasks [20]. Narcissism is exemplified by grandiosity, entitlement, dominance, and superiority [21], and has been linked with aggression in response to threats to self-esteem or status [22], increased prejudice towards immigrants [23], and unethical behaviour among CEOs [24]. Finally, sadism is distinguished by pleasure from victim suffering [25], interest in violent entertainment [26,27], and a willingness to pay a price to hurt others [1].

Each Dark Tetrad trait may also be divided into narrower facets. For example, contemporary research suggests Machiavellianism is a two-dimensional construct involving both views and tactics, captured by the Two-Dimensional Machiavellianism Scale (TDMS) [28]. Individuals scoring high on the views dimension tend to hold a pessimistic opinion of humanity, seeing others as inherently untrustworthy or vulnerable to exploitation. Machiavellian tactics reflect an individual’s willingness to strategically and rationally engage in traditionally immoral behaviour to achieve their goals; individuals who score high on this dimension tend to be exploitative and callous, yet cooperative and agreeable depending on which behaviours are advantageous in a particular context. Similarly, distinct narcissism facets of grandiosity and vulnerability may be measured by the Brief Pathological Narcissism Inventory (B-PNI) [29]. Grandiose narcissism is illustrated by traits of grandiosity, aggression, dominance, inflated self-esteem, and entitlement; whereas vulnerable narcissism is differentiated by an insecure and defensive grandiosity that masks feelings of inadequacy and incompetence, as well as emotional instability, introversion, and oscillation between feelings of superiority and inferiority [30,31].

Traditional measures of the Dark Tetrad are intentionally broad to maximize their coverage. A consequence of broad bandwidth, however, is a tendency for measures to overlap, such that individuals who score high on measures of sadism, for example, will also score high on measures of psychopathy and Machiavellianism [7,32,33]. The overlap can obscure each trait’s distinct effects. To better differentiate psychopathy, narcissism, Machiavellianism, and sadism, a four-factor instrument, the Short Dark Tetrad (SD4) [7], was developed to minimize the overlap between these socially aversive personalities by emphasizing their unique components.

### Behavioural inhibition and approach systems

Differences among the Dark Tetrad traits and their respective facets may translate into meaningful differences in behaviour. Of particular relevance to the present study, individuals with different dark inclinations may differ in behavioural inhibition and approach. Gray’s Reinforcement Sensitivity Theory [34,35] proposes two distinct motivational systems involved in the regulation of behaviour: the Behavioural Inhibition System (BIS), responsible for controlling the experience of anxiety in response to anxiety-relevant stimuli, and the Behavioural Activation System (BAS), which controls appetitive motivation. BIS activation is said to cause inhibition of an individual’s movement toward goals, whereas BAS activation causes mainly movement toward goals, whether prosocial or antisocial. Carver and White [36] developed a questionnaire measure of dispositional BIS and BAS activation levels. They maintained that within a given population, all possible combinations of high and low BIS and BAS activations should be observed. Consistent with this assertion, the Big Five personality dimensions are associated with different BIS/BAS activation patterns, reflecting the distinct motivational qualities of each trait domain [37]. The Dark Tetrad may likewise be associated with interpretable BIS/BAS activation patterns. However, unlike the Big Five, which are conceptualized as orthogonal or independent personality factors [38], the Dark Tetrad traits are conceptualized as oblique or correlated factors [7], which may result in overlapping BIS/BAS activation patterns that are more challenging to disentangle.

A meta-analysis conducted by Włodarska et al. [39] provides evidence for a motivational profile that is common to most Dark Tetrad traits, specifically a combination of weak BIS and strong BAS that favors approach behaviour. Narcissism is generally considered the “brightest” of the Dark Tetrad traits because it tends to be associated with more positive outcomes than “darker” traits like psychopathy [40]. Despite this distinction, Włodarska et al.’s meta-analysis showed that grandiose narcissism and psychopathy were associated with a similar pattern of reduced BIS activation and increased BAS activation [39], and can thus be understood as high-approach/low-avoidance traits [cf. 41]. Sadism is also a high-approach/low-avoidance trait [3], but the association pattern for Machiavellianism is less clear [39]. These associations were demonstrated with questionnaire measures of dispositional BIS and BAS activation levels. What has not been determined, however, is whether these proposed relationships between the Dark Tetrad and behavioural inhibition and approach systems are accurately reflected in approach-avoidance paradigms assessing actual behaviour.

The literature suggests that approach-avoidance behaviour is partly mediated by anxiety, with greater anxiety influencing any measure to the extent that it is governed by the BIS. This hypothesis has received substantial support using approach-avoidance conflict tasks (AACT). Anxiety-provoking situations in real life are often mixed-outcome situations, where avoidance of an aversive outcome may only occur at the cost of losing a competing reward. Hoppe et al. [42] provide the example of a socially anxious individual who avoids dating. In doing so, the individual reduces the risk of rejection (avoidance of an aversive outcome) but never disconfirms their belief that they will be rejected (loss of potential reward). To emulate this real-world conflict in their approach-avoidance task, Hoppe et al. adapted a computer task [43] where participants could earn points for choosing a skull icon, which was then followed by a negative image, but no points for choosing the smiley face icon, which was then followed by a positive image. They reported that individuals who scored higher on measures of anxiety tended to exhibit increased levels of costly avoidance. That is, they required more points to approach an aversive stimulus, the skull. Exchangeable for nothing within the experiment, the points presumably acted like social or non-social primary reinforcers in the reward circuitry of the brain [44,45]. Similar AACT paradigms have uncovered associations between major depressive disorder (MDD) and reduced reward sensitivity [46], and increased costly avoidance in individuals with post-traumatic stress disorder [47].

Building on these previous findings, the present studies used a reverse translational approach [44] to investigate approach-avoidance conflict with computer tasks that were administered either in lab (Studies 1 and 2) or online (Studies 3 and 4). We were particularly interested in testing the possibility that AACT behaviour varies across the traits of the Dark Tetrad. Studies 1 and 2 consisted of an initial online questionnaire battery, followed several months later by an in-person AACT. Studies 3 and 4 were gender-balanced online studies consisting of two questionnaires, followed by an AACT, which ended with a viewing-time task (VTT). Our hypotheses were informed by the Dark Tetrad model [9] and grounded in Gray’s Reinforcement Sensitivity Theory [34,35]. Across these studies, we expected most Dark Tetrad traits to predict greater approach toward negative stimuli due to a lessened BIS (e.g., diminished sensitivity to cues of punishment like a negative icon) and heightened BAS (e.g., higher responsiveness to point-based incentives for approaching the negative stimulus). Differences among the Dark Tetrad were probed as exploratory analyses. An a-priori power analysis indicated that a sample size of 193 participants was necessary to detect a small correlation of ρ = 0.20 with power (1 – β) = 0.80. This result guided our sample size determination for each study.

## Study 1

**Fig 1:**
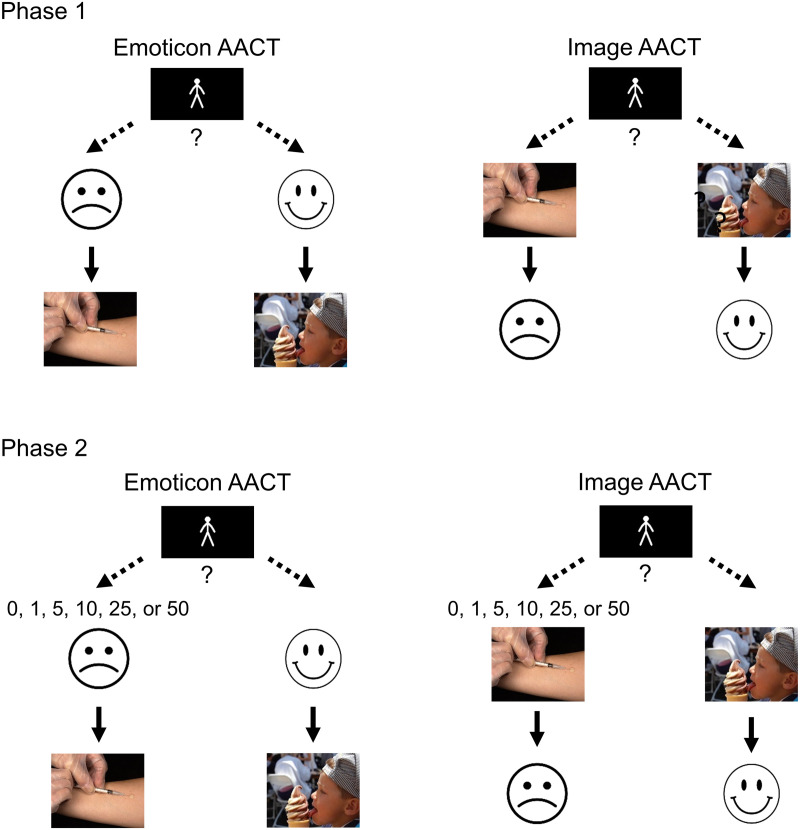
Approach-Avoidance Conflict Task (AACT). Participants chose between two options by pressing either the right or left arrow key on the computer keyboard, which caused the stick figure to walk toward the chosen emoticon (Emoticon AACT; Studies 1, 3, and 4) or image (Image AACT; Study 2). In Phase 2, one of six point values was displayed randomly above the negative emoticon/image at the time of choice. Outcomes were displayed for 4 s immediately after the stick figure reached its destination. Images shown in the figure (shot1, dessert5) come from the Open Affective Standardized Image Set.

The AACT used in Study 1 (Emoticon AACT) consisted of 24 trials in two phases adapted from Hoppe et al. [42]. Fig 1 provides an overview of the task. In Phase 1, participants were told to imagine they were the stick-figure manikin depicted on the computer screen [48,49], and they then made a choice by a single press on the right or left arrow key on the keyboard. Depending on their decision, the participant watched the manikin move in the chosen direction, and then they were shown a randomly selected positive or negative image when the manikin reached the smiley or frowny-face destination, respectively. In Phase 2, participants were presented with a conflict: A varying number of points could now be earned for choosing the frowny icon, but not for choosing the smiley icon. Participants who wished to view the positive image could continue to do so, but would incur a cost of not earning points.

We expected that participants exhibiting high-approach/low-avoidance aversive traits—notably sadism, psychopathy, and grandiose narcissism [3,39]—should demonstrate greater motivation to approach the frowny face. This tendency should be greater in Phase 2 than Phase 1 of the AACT because the points act as an additional source of motivation. Additionally, we expected those with tactical Machiavellian tendencies to be motivated to earn points, and TDMS subscale scores should be correlated with accepting fewer points to approach the frowny icon in Phase 2. By contrast, those with high anxiety sensitivity should have relatively high BIS activations, leading to costly avoidance on the AACT. A measure of anxiety sensitivity was included to confirm the AACT was sensitive to anxiety-related personality variables, particularly physical anxiety [44]. Lastly, we included measures of empathy to provide insight into the Dark Tetrad correlation patterns.

### Method

#### Participants.

A total of 197 participants (153 women, 42 men, 2 non-binary) were recruited from the introductory psychology subject pool at the University of Winnipeg (recruitment period: 30-Oct-2023–2-Feb-2024). They received partial class credit as thanks for their participation. Online administration of the questionnaire battery was conducted using Qualtrics (Qualtrics, Provo, UT), approximately 2–4 months before their lab visit. Administration of the Emoticon AACT occurred in-lab on a laptop. Ethics approval was obtained from the Research and Ethics Committee of the Department of Psychology at the University of Winnipeg.

#### Materials.

***Questionnaires:*** **Short Dark Tetrad (SD4).** The SD4 measures subclinical levels of Machiavellianism (e.g., “It’s not wise to let people know your secrets”), narcissism (e.g., “I’m likely to become a future star in some area”), psychopathy (e.g., “People often say I’m out of control”), and sadism (e.g., “Watching a fist-fight excites me”) [7]. The 28 items are rated on a Likert scale from 1 (strongly disagree) to 5 (strongly agree). The SD4 was designed to maximize the separation of Dark Tetrad members, measuring them more narrowly to reduce overlap and increase fidelity.

**Two-Dimensional Machiavellianism Scale (TDMS).** This self-report measure has separate subscales for Machiavellian views and tactics [28]. Machiavellian worldviews reflect a pessimistic view of humanity as inherently untrustworthy (e.g., “When people do something nice for me they really have another agenda”), whereas Machiavellian tactics reflect an individual’s willingness to partake in strategic immoral behaviour to achieve their goals (e.g., “It is sometimes necessary for me to mislead others to get things done”). Items are rated on a Likert scale from 1 (strongly disagree) to 7 (strongly agree), with six items contributing to each dimension.

**Brief Pathological Narcissism Inventory (B-PNI).** The B-PNI is a shortened version of the PNI [50], composed of 28 items to measure the grandiose (e.g., “I often fantasize about performing heroic deeds”) and vulnerable (e.g., “When people don’t notice me, I start to feel bad about myself”) dimensions of pathological narcissism [29]. Participants indicate how much the statements are typical of them using a Likert scale from 1 (not at all like me) to 6 (very much like me).

**Triarchic Psychopathy Measure (TriPM).** The Triarchic Psychopathy Measure is a 58-item, multidimensional questionnaire that characterizes the symptomatic components of psychopathy in terms of disinhibition, boldness, and meanness [51]. Disinhibition refers to the general propensity toward problems of impulse control (e.g., “I jump into things without thinking”); boldness is concerned with social dominance, emotional resiliency, and sensation-seeking (e.g., “I’m a born leader”); and meanness is related to callousness, antagonism, and arrogance (e.g., “I don’t have much sympathy for people”). Participants are asked to rate their agreement with various statements on a Likert scale from 1 (false) to 4 (true).

**Comprehensive Assessment of Sadistic Tendencies (CAST-12).** The CAST-12 assesses chronic tendencies towards sadistic behaviours using a short, 12 item scale [52]. This measure makes a distinction between the enjoyment of harming others physically (direct physical sadism; e.g., “I enjoy physically hurting people”) or verbally (direct verbal sadism; e.g., “I enjoy making jokes at the expense of others”), versus the enjoyment of observing others being harmed (vicarious sadism, e.g., “I sometimes replay my favorite scenes from gory slasher films”). Participants are asked to rate the extent to which they agree with various statements about themselves using a 5-point Likert scale from 1 (strongly disagree) to 5 (strongly agree).

**Affective and Cognitive Measure of Empathy (ACME).** This instrument was designed to measure three aspects of empathy: affective resonance, cognitive empathy, and affective dissonance [4]. Affective resonance involves sharing the feelings of another (e.g., “I get excited to give someone a gift that I think they will enjoy”); cognitive empathy is the ability to accurately decode and understand another’s emotion (e.g., “I usually understand why people feel the way they do”); whereas affective dissonance refers to the experience of an emotional response that does not fit the objective circumstance, and is contradictory to the emotions of others (e.g., “When my friends are having a good time I often get angry”). Affective dissonance is observed at low levels of empathy, and is thus reverse coded to serve as an empathy measure. The 36 items on this scale are rated using a Likert scale from 1 (disagree strongly) to 5 (agree strongly).

**Behavioural Inhibition System/Behavioural Activation System (BIS/BAS) Scales.** The BIS/BAS measures aversive and appetitive motivation [36]. The scale has 24 items with a single BIS measure (motivation to avoid negative outcomes) and three BAS measures (motivations to approach). The three BAS measures are drive, which contains items pertaining to the persistent pursuit of desired goals (e.g., “I go out of my way to get things I want”); fun seeking, with items reflecting a desire for new rewards and a willingness to approach a potentially rewarding event on the spur of the moment (e.g., “I’m always willing to try something new if I think it will be fun”); and reward responsiveness, which has items focusing on positive responses to the occurrence or anticipation of a reward (e.g., “When I get something I want, I feel excited and energized”). Statements are rated on a Likert scale from 1 (very true for me) to 4 (very false for me).

**Anxiety Sensitivity Index (ASI-3).** The ASI-3 focuses on apprehension about the symptoms of anxiety [53]. This three-factor scale has 18 items, six items each measuring physical symptoms (e.g., “When my stomach is upset, I worry that I might be seriously ill”), cognitive symptoms (“When my mind goes blank, I worry there is something terribly wrong with me”), and social anxiety symptoms (e.g., “I worry that other people will notice my anxiety”). Participants use a Likert scale from 1 (very little) to 5 (very much) to rate how they thought they might feel if they had various experiences.

***Images:*** Images were obtained from several photo databases for academic research in psychology, namely the DIsgust-RelaTed-Images [54], EmoMadrid [55], Nencki Affective Picture System [56], and the Open Affective Standardized Image Set [57]. Half (48) of these images were positive in valence, depicting joyful faces, appealing landscapes, and fun activities (see S1A Table in S1 File). The remaining 48 images were negative in valence, depicting animal and human suffering, threats (guns), gory injuries, garbage and body products, and death.

#### Procedure.

Participants were led to the experimental room and asked to take a seat in front of the computer. After reading and indicating their written agreement with the consent form, participants completed the Emoticon AACT computer task. The Emoticon AACT consisted of 48 trials in total, divided into two blocks of 24 trials programmed in PsychoPy [58]. Participants were prompted to press the spacebar to begin the first block of 24 trials (Phase 1). On each trial, the participants were presented with two icons, a smiley face and a frowny face. The icons were shown on the right and left sides of the screen, the frowny icon on one side and the smiley icon on the other side, with their placement determined randomly on each trial. A stick-figure manikin appeared in the centre of the screen, equidistant from both stimuli. Participants were instructed the “stick-figure manikin in the centre of the screen represents YOU” (see S1 File). Participants pressed the ‘right’ or ‘left’ arrow key on the keyboard to move the manikin toward the icon of their choice. Their response was followed by a 2.7 s animation of the stick-figure manikin walking in the direction of the input (right or left). If the participant chose the smiley icon, they were shown a positive image for 4.0 s. If they chose the frowny icon, they were shown a negative image for 4.0 s. The positive and negative images were randomly drawn on each trial without replacement. There was a 500 ms intertrial interval (ITI), followed by the presentation of a 300 ms fixation dot before each trial. Reaction time and choice were measured for each trial.

Following the completion of the first block of 24 trials, the participants were prompted to press the spacebar on the keyboard to begin the second block (Phase 2). The second 24-trial block proceeded in a similar fashion, except the addition of points, which could now be earned for approaching the frowny icon. Each trial was randomly attributed a point value, varying from 0, 1, 5, 10, 25, or 50 points, with the point value displayed in text above the frowny icon on the screen. Participants who chose to move the manikin towards the frowny icon were shown a negative image for 4.0 s, as in Phase 1, but were also rewarded with a variable number of points. Participants who chose to approach the smiley icon were shown a positive image for 4.0 s, but were not awarded points. A feedback message indicating the number of points earned from that trial and the total number of points earned so far was also displayed alongside the image. Reaction time, choice, number of points available, and total number of points earned were measured for each trial. A link to a demo of the Emoticon AACT procedure can be found in S1 File.

***Data analysis:*** To operationalize behavioural approach choices directed at negative stimuli, the dependent variable “switch point” was used to characterize any change in Phase 2 caused by varying the number of available points on a trial. Switch point was defined as the magnitude of the point value required to motivate an individual to approach the frowny icon, thus ‘switching’ from viewing the positive image to viewing the negative image. If a participant approached the frowny icon equally or more often than the smiley icon for 0 or 1 points, they were assigned a switch point value of 1. If 5 or 10 points were required to entice them to approach the frowny icon equally or more often than the smiley icon, they were assigned a switch point value of 2. If 25 or 50 points were required to entice them to approach the frowny icon equally or more often than the smiley icon, they were assigned a switch point value of 3, and, if they could not be enticed with points to approach the frowny icon equally or more often than the smiley icon no matter the number of points available, they were assigned a switch point value of 4. As a result of this operationalization, individuals who are motivated to approach the frowny face and view a negative image for fewer points will thus exhibit a lower switch point value. The percentage of trials in which participants approached the frowny face in Phases 1 and Phase 2 of the AACT served as the global (irrespective of points) dependent variable.

### Results

Bivariate correlations between Study 1 predictors and Emoticon AACT behaviour as measured by negative approach and switch point are shown in Table 1. That table also contains descriptive statistics for the predictor variables. Participants approached the frowny face in Phase 1 (Phase 1 negative approach) on 42.5% of trials (*SD* = 0.26), and in Phase 2 (Phase 2 negative approach) on 60.2% of trials (*SD* = 0.29). Mean response latency did not differ between Phase 2 and Phase 1, *M*_*diff*_ = 0.02, *t*(196) = −0.29, *p* = .769, η^2^ = 0.000. Switch point was closely related to Phase 2 negative approach, *r*(195) = − .912, *p* < .001, with these alternative dependent variables very strongly and inversely correlated in Study 1. As in Phase 1, nearly half the participants (42.1%) needed no or little inducement to choose the negative icon and had a switch point of 1, indicating that they consistently approached the frowny icon for 0 or 1 points. Of the 83 participants with a switch point of 1, 52 approached the frowny face for a single point, but did not approach for 0 points, and 31 approached for both 0 and 1 points. Among these 83 participants, the distinction between approaching for 1 point versus approaching for both 0 and 1 point was not associated with any Dark Tetrad tendencies.

**Table 1:** Study 1 personality measure descriptive statistics and bivariate correlations with AACT behaviour.

Scale	*M*	*SD*	Range	α	P1 NA	P2 NA	SP
Gender: M = 1; W = 2					−.105	−.147*	.143*
SD4							
Machiavellianism	3.47	.716	1.86–5.00	.78	.080	.147*	−.145*
Narcissism	2.97	.721	1.00–4.57	.76	−.008	.034	−.061
Psychopathy	1.87	.748	1.00–5.00	.83	−.031	.064	−.033
Sadism	2.37	.912	1.00–4.57	.83	.017	.131	−.101
TDMS							
Views	3.85	.969	1.00–6.83	.69	.090	.108	−.098
Tactics	3.22	1.152	1.00–7.00	.79	.086	.210**	−.191**
B-PNI							
Grandiose	3.69	.872	1.00–5.92	.83	−.120	.075	−.111
Vulnerable	3.30	.951	1.00–6.00	.88	−.053	−.023	−.017
TriPM							
Boldness	2.40	.408	1.37–3.58	.77	.053	.183**	−.194**
Meanness	1.72	.440	1.00–3.21	.85	.081	.179*	−.148*
Disinhibition	1.94	.360	1.20–3.00	.74	.082	.071	−.056
CAST-12							
Total	1.72	.666	1.00–4.33	.87	.146*	.136	−.146*
Direct Verbal	2.02	.951	1.00–5.00	.82	.091	.102	−.114
Direct Physical	1.28	.552	1.00–3.50	.83	.178*	.119	−.144*
Vicarious	1.86	.968	1.00–5.00	.83	.111	.111	−.107
ACME							
Resonance	4.12	.558	2.00–5.00	.80	−.185**	−.202**	.155*
Cognitive	3.65	.669	1.25–5.00	.89	−.069	.000	−.039
Dissonance (lack)	4.14	.658	2.08–5.00	.78	−.106	−.157*	.140*
BIS/BAS							
BAS Drive	2.71	.606	1.00–4.00	.76	−.065	.149*	−.165*
BAS Fun Seeking	3.06	.526	1.25–4.00	.60	−.171*	.076	−.074
BAS Reward	3.48	.436	1.20–4.00	.69	−.131	−.008	.005
BIS	3.21	.472	1.71–4.00	.71	−.129	−.098	.085
ASI-3							
Cognitive	2.60	1.006	1.00–5.00	.86	−.029	−.068	.088
Social	2.93	.823	1.17–5.00	.70	−.057	−.063	.045
Physical	2.48	.956	1.00–5.00	.84	−.046	−.172*	.163*

*Note: n* = 197. P1 NA = Phase 1 negative approach; P2 NA = Phase 2 negative approach; SP = switch point; SD4 = Short Dark Tetrad; TDMS = Two-Dimensional Machiavellianism Scale; B-PNI = Brief Pathological Narcissism Inventory; TriPM = Triarchic Psychopathy Measure; CAST-12 = Comprehensive Assessment of Sadistic Tendencies; ACME = Affective and Cognitive Measure of Empathy; ASI-3 = Anxiety Sensitivity Index-3; BIS/BAS = Behavioural Inhibition System/Behavioural Activation (Approach) System.

*p < .05, **p < .01.

Bivariate correlations (see Table 1) revealed significant associations between Emoticon AACT behaviour in Phase 2 with sadistic, psychopathic, and Machiavellian—but not narcissistic—tendencies. Of these, Machiavellianism was most consistently associated with Emoticon AACT behaviour. SD4 Machiavellianism was associated with lower switch point and higher Phase 2 negative approach, as was TDMS Machiavellian tactics but not worldview. Switch point was positively associated with two facets of empathy, ACME affective dissonance and affective resonance. (Note, ACME affective dissonance and resonance are positively correlated with each other because affective dissonance is reverse scored with high scores representing lower levels [lack] of dissonant emotions). Scores on the ACME affective dissonance scale were strongly inversely correlated with SD4 sadism, *r*(193) = − .603, *p* < .001, as shown in S2 Table in S1 File, although only affective dissonance reached statistical significance with switch point and Phase 2 negative approach as shown in Table 1. TriPM boldness and meanness also reached significance, the latter being the psychopathic trait most closely related to SD4 sadism, *r*(192) =.576, *p* < .001, as shown in S2 Table in S1 File. No significant bivariate correlations were observed between measures of narcissism and Emoticon AACT behaviour.

The Dark Tetrad have empirical overlap that requires consideration, so a multiple regression analysis was performed in an attempt to determine if one of these aversive traits could predict switch point when controlling for the others. Thus, we entered the four SD4 variables as predictors of switch point, and found that no Dark Tetrad traits uniquely predicted participants’ Emoticon AACT behaviour (Table 2). Similar results were found with Phase 2 negative approach (Table 2) and when gender was also entered as a predictor (S3 Table in S1 File).

**Table 2:** Studies 1 and 2 multiple linear regressions predicting switch point and Phase 2 negative approach from the Dark Tetrad during the AACT.

Study	Variable	*B*	*SE*	β	*Robust* *SE*	*t*	*p*
Study 1	Switch Point						
	SD4 Machiavellianism	−.169	.122	−.108	.122	−1.384	.168
	SD4 Narcissism	−.074	.115	−.048	.118	−0.626	.532
	SD4 Psychopathy	−.011	.122	−.007	.145	−0.073	.942
	SD4 Sadism	−.072	.103	−.059	.111	−0.652	.515
	Phase 2 Negative Approach						
	SD4 Machiavellianism	.041	.031	.101	.031	1.329	.185
	SD4 Narcissism	.005	.029	.013	.030	0.172	.863
	SD4 Psychopathy	.017	.031	.044	.035	0.482	.630
	SD4 Sadism	.024	.026	.076	.027	0.883	.378
Study 2	Switch Point						
	SD4 Machiavellianism	−.191	.140	−.102	.147	−1.298	.196
	SD4 Narcissism	.222	.121	.141	.129	1.719	.087
	SD4 Psychopathy	.141	.136	.085	.127	1.105	.271
	SD4 Sadism	−.232	.109	−.174	.111	−2.095	.037*
	Phase 2 Negative Approach						
	SD4 Machiavellianism	.064	.038	.127	.038	1.676	.096
	SD4 Narcissism	−.052	.033	−.121	.036	−1.440	.152
	SD4 Psychopathy	−.032	.037	−.070	.035	−0.900	.369
	SD4 Sadism	.054	.029	.151	.031	1.761	.080

*Note*: Valid *n*’s = 194 (Study 1) and 191 (Study 2), SD4 = Short Dark Tetrad, *t* and *p* on the basis of the robust *SE.*

*p < .05.

There were also a number of significant associations involving BIS/BAS sensitivities. BAS drive was significantly associated with both low switch point and high Phase 2 negative approach. BAS fun seeking was negatively correlated with Phase 1 negative approach. One ASI-3 facet, physical anxiety, showed a significant positive association with switch point, suggesting participants prone to physical anxiety needed more points to motivate approach towards the frowny icon.

### Discussion

The Emoticon AACT used in Study 1 revealed associations between approach/avoidance choices and Dark Tetrad traits. Facets of sadism, psychopathy, and Machiavellianism—but not narcissism—were all associated with choosing the frowny icon, though likely for different reasons. Sadists may have chosen the frowny icon for fewer points because they find negative images subjectively appealing and/or because they dislike positive images [2,3]. Machiavellians likely approached because their motivation to earn points outweighed the disturbance they felt upon being shown a negative image. This assertion is supported by previous research which found that individuals who score high on measures of Machiavellian tactics are highly motivated to seek reward regardless of potential consequences [20]. TriPM boldness correlates reliably with most facets of extraversion and related constructs (social potency, well-being, and social closeness), as well as most facets of emotional stability/low neuroticism (except hostility), and low stress reactivity [59]. As such, potential motivations for approaching the frowny icon in participants with bold psychopathic tendencies may include a desire to draw attention to themselves or a propensity for engaging in risky behaviour [60].

Sadism and psychopathy are high approach/low avoidance traits [3,39], and it was hypothesized that these general tendencies would motivate approach toward the negative images during the AACT, especially if there was an external reward (points) available for doing so. The significant associations between BAS sensitivities and various measures of task behaviour support this assertion. Participants with high BAS drive sensitivities were more likely to approach the negative image in Phase 2 than in Phase 1, which is consistent with the idea that BAS drive is associated with persistent pursuit of desired goals [36]. BAS fun seeking was negatively associated with Phase 1 negative approach, suggesting that participants with fun-seeking tendencies consider viewing negative images to be decidedly *not* fun, and not worth approaching when there is no external motivator (points). Additionally, the positive images included many fun images of peaceful scenes or playful interactions.

It was also hypothesized that individuals with higher levels of anxiety would require more points to motivate approach toward the negative image [42], a prediction that was supported by the positive association between ASI-3 physical anxiety and switch point in the present task. Thus, the Emoticon AACT task was sensitive to individual differences driving behaviour in the opposite direction as socially aversive personality. According to the two-process theory of avoidance [61], when an aversive event is preceded by a warning stimulus, those two stimuli become paired through classical conditioning. According to this theory, the frowny icon should have become further negatively valenced by being paired with the negative images, above and beyond its incoming negative value, also established through past experience. Physical anxiety may gradually displace curiosity over trials as the participants learn from the pairings. Subsequent responses that avoid the warning stimulus (i.e., moving the manikin toward the smiley face) are then reinforced, because the response causes a reduction in anxiety. The associations between anxiety measures and switch point across Study 1 are consistent with this well-regarded model, which has since been modified considerably [62].

Neither SD4 narcissism, nor B-PNI grandiose or vulnerable narcissism were significantly associated with task choices in the present study. Grandiose narcissism has been conceptualized as a high-approach/low-avoidance trait [39], prompting the hypothesis that grandiose narcissists would decide to approach the negative image for fewer points. This hypothesis was not supported by the present study.

No Dark Tetrad trait uniquely predicted switch point, indicating that while the Emoticon AACT captures high-approach/low-avoidance malevolent tendencies more generally, it cannot sufficiently tease them apart. In Study 2, we made alterations to the AACT in an attempt to emphasize the aversive content of the negative images to capture sadistic tendencies over and above the other Dark Tetrad traits.

## Study 2

The Image AACT used in Study 2 was identical to the Emoticon AACT except in one key area: The arrangement of stimuli was changed such that the positive and negative images were shown next to the stick-figure manikin, and depending on which image the participant chose, they were shown a smiley or frowny icon, respectively (see Fig 1). With this new arrangement, we expected that only participants with sadistic tendencies would choose the negative image for fewer points, given that sadism is correlated with the reduced subjective appeal of some types of positive images and the increased subjective appeal of some types of negative images [2,3,63]. The image layout reduces the role for anticipatory anxiety because the negative and positive stimuli are in view on the screen on all trials while participants made their decision. Experiencing the frowny face may simply indicate to the participant they made a socially unacceptable choice. In Phase 2, that socially unacceptable choice also delivered a varying number of points, and the question of interest was how many points would be required to get some people to choose the negative images.

### Method

#### Participants.

An additional 191 participants (145 women, 40 men, 6 non-binary) were recruited from the introductory psychology subject pool at the University of Winnipeg (recruitment period: 24-Nov-2022–7-Feb-2023), receiving partial class credit for their participation. Due to the limitations of our participant pool, the sample size did not quite reach the number determined by our power analysis (*n* = 193), but the discrepancy was minor. Study administration methods were identical to Study 1, and all participants provided written informed consent. Ethics approval was obtained from the Research and Ethics Committee of the Department of Psychology at the University of Winnipeg.

#### Materials.

The questionnaire battery administered in the present study was identical to Study 1.

#### Procedure.

The images used in the study, the points structure, and the manikin animation remained identical to Study 1. An alteration was made to the AACT that affected the order in which the icons and images were shown. In Study 2 (Image AACT), the arrangement was changed so the positive and negative images were placed next to the manikin stick-figure, and depending on which image the participant chose, they were shown either a smiley or frowny icon. This new arrangement resulted in the images being shown while the participants’ made their choices, with the stick-figure avatar moving toward or away from the positive or negative images. A slight adjustment was also made to the Phase 1 instructions in the present study, with participants being instructed to approach the positive image in Phase 1, and then being given the choice to continue approaching the positive image in Phase 2, or to approach the negative image instead and earn points. These changes were implemented with the intent to create an AACT that could tease apart sadistic tendencies from other high approach/low avoidance Dark Tetrad traits by showcasing the content of the negative images, which may be pleasing to people high in sadism.

### Results

Participants approached the negative image in Phase 1 (Phase 1 negative approach) on 2.7% of trials (*SD* = 0.04), and in Phase 2 (Phase 2 negative approach) on 31.8% of trials (*SD* = 0.32). Mean response latency was greater in Phase 2 than Phase 1, *M*_*diff*_ = 0.47, *t*(190) = 8.19, *p* < .001, η^2^ = 0.23. Switch point was again closely and inversely related to Phase 2 negative approach, *r*(189) = − .962, *p* < .001. A slight majority of participants (51.8%) never switched, and were thus attributed a switch point of 4, with the remaining participants distributed evenly across switch point 1–3. Of the 35 participants with a switch point of 1, 17 approached the negative image for a single point, but did not approach for 0 points, and 18 approached for both 1 point and 0 points. As was the case in Study 1, the distinction between approaching for 1 point versus approaching for both 0 and 1 point was not associated with any Dark Tetrad tendencies. Overall, across Phase 1 and Phase 2, participants approached the negative image on far fewer trials during the present study than participants approached the frowny icon in Study 1. S4 Table in S1 File presents the correlations among the predictor variables.

Bivariate correlations between Study 2 predictors and Image AACT choices as measured by switch point and negative approach are shown in Table 3. Although bivariate correlations revealed no significant associations between any of the SD4 subscales and Phase 2 negative approach or switch point, there was a significant positive correlation between CAST-12 vicarious sadism and Phase 2 negative approach. Fewer points were also required on our switch point variable for those higher on vicarious sadism. In addition, TriPM meanness was positively associated with Phase 2 negative approach, whereas TriPM boldness was positively associated with switch point. The only other measures that revealed associations with task performance were facets of empathy (ACME). Affective dissonance was negatively associated with Phase 1 negative approach, as it had been in Study 1.

**Table 3:** Study 2 personality measure descriptive statistics and bivariate correlations with AACT behaviour.

Scale	*M*	*SD*	Range	α	P1 NA	P2 NA	SP
Gender: M = 1; W = 2					.018	−.109	.073
SD4							
Machiavellianism	3.43	.632	1.29–5.00	.72	−.085	.130	−.105
Narcissism	2.80	.753	1.00–5.00	.82	−.038	−.082	.106
Psychopathy	1.86	.712	1.00–4.43	.80	.150*	−.026	.040
Sadism	2.34	.888	1.00–4.71	.82	.056	.123	−.129
TDMS							
Views	3.77	.947	1.00–6.83	.69	.063	.035	.011
Tactics	3.30	1.096	1.00–6.33	.75	−.023	.095	−.069
B-PNI							
Grandiose	3.58	.854	1.33–5.75	.84	−.040	.059	−.038
Vulnerable	3.16	.886	1.44–5.75	.87	−.005	.081	−.095
TriPM							
Boldness	2.42	.456	1.26–3.68	.83	−.016	−.132	.182*
Meanness	1.72	.417	1.05–3.00	.85	.044	.150*	−.110
Disinhibition	1.97	.441	1.10–3.35	.83	.155*	.050	−.039
CAST-12							
Total	1.78	.680	1.00–4.75	.87	.045	.150*	−.128
Direct Verbal	2.08	.913	1.00–4.75	.78	.033	.126	−.100
Direct Physical	1.38	.686	1.00–5.00	.89	.054	.064	−.045
Vicarious	1.90	.957	1.00–5.00	.84	.025	.154*	−.145*
ACME							
Resonance	4.21	.576	2.25–5.00	.86	−.077	−.169*	.119
Cognitive	3.72	.616	2.25–5.00	.88	−.121	−.171*	.166*
Dissonance (lack)	4.13	.708	2.00–5.00	.90	−.147*	−.165*	.146*
BIS/BAS							
BAS Drive	2.71	.576	1.00–4.00	.72	.022	.055	−.011
BAS Fun Seeking	3.04	.547	1.75–4.00	.65	−.013	−.025	.046
BAS Reward	3.51	.434	2.20–4.00	.70	−.070	.007	.006
BIS	3.20	.487	2.00–4.00	.74	−.001	.010	−.047
ASI-3							
Social	1.90	.925	0.00–4.00	.78	.008	.106	−.110
Cognitive	1.41	1.048	0.00–4.00	.88	.037	.118	−.098
Physical	1.49	1.051	0.00–4.00	.87	.093	.091	−.079

*Note*: *n* = 191. P1 NA = Phase 1 negative approach; P2 NA = Phase 2 negative approach; SP = switch point; SD4 = Short Dark Tetrad; TDMS = Two-Dimensional Machiavellianism Scale; B-PNI = Brief Pathological Narcissism Inventory; TriPM = Triarchic Psychopathy Measure; CAST-12 = Comprehensive Assessment of Sadistic Tendencies; ACME = Affective and Cognitive Measure of Empathy; ASI-3 = Anxiety Sensitivity Index-3; BIS/BAS = Behavioural Inhibition System/Behavioural Activation (Approach) System. *p < .05.

Entering the SD4 subscales as predictor variables, and switch point as the outcome variable in a multiple linear regression, SD4 sadism emerged as the only unique predictor (see Table 2). However, with gender included, this relationship became non-significant, *p* = .104 (S5 Table in S1 File). No unique associations were uncovered with Phase 2 negative approach, although SD4 sadism approached significance (Table 2).

### Discussion

With the new arrangement of stimuli where the images could be seen, the addition of points above the negative image enticed cruel individuals who enjoy watching violence (CAST-12 vicarious sadism and TriPM meanness) and those with dissonant emotional tendencies (ACME affective dissonance) to approach the negative images for fewer points. Measures that were unrelated to sadism or dissonant emotions failed to predict AACT choices. Psychopathy has been linked to callousness and affective desensitization [64], but these tendencies do not necessarily result in positive emotional responses to negative images or negative emotional responses to positive images. Machiavellianism is characterized by cynicism and manipulation [28], but not to unusual emotional reactions to images in the absence of anxiety. Narcissism, measured by the SD4 or B-PNI [29], also was not associated with AACT variables in Study 2.

Like Study 1, TriPM meanness showed a positive correlation with Phase 2 negative approach, an association that corroborates the sadism result, given the strong conceptual and empirical overlap between SD4 sadism and TriPM meanness (S4 Table in S1 File). The result of our regression analysis supports these trends—SD4 everyday sadism predicted switch point controlling for psychopathy, Machiavellianism, and narcissism. This finding, alongside the significant bivariate correlations between ACME dissonance and our two dependent variables (P2 NA and SP), suggests that choices depend on subjective appeal of the images—whether the content of the image elicits a pleasant or unpleasant emotional response for that individual—not the low physical anxiety associated with the Dark Tetrad, as might arguably be the case in Study 1 (Emoticon AACT). It is possible that people high in sadism were drawn toward the negative content. However, only a handful of the aversive images presented during the AACT depict scenes of human suffering and violence, the types of stimuli that highly sadistic individuals find intrinsically rewarding [2,3]. It is possible, and perhaps even more likely, that the tendency to feel negative emotions when viewing positive images [2,3] drove them away from the positive images and toward the negative images.

The gender distributions in both Studies 1 and 2 present a substantial limitation, with 75–80% of the sample reporting their gender identity as “woman”. Given the historical tendency for men to report higher levels of Dark Tetrad traits [7], having a relative lack of men in these samples could have affected the strength of our results. This is an inherent limitation with using non-balanced samples, and we addressed this shortcoming by using gender-balanced online samples in Studies 3 and 4.

## Studies 3 and 4

Studies 3 and 4 used an identical Emoticon AACT as Study 1 with gender-balanced samples recruited through Prolific. We chose the SD4 and ACME to predict AACT performance, and introduced an additional viewing-time task (VTT). This task, modified from Parsons et al. [65], included positive and negative valenced images rather than images of infants. The VTT is based on the theory that participants will work to view images they find affectively rewarding [65,66]. These VTT data should tell us how participants with socially aversive traits, and in particular sadism and dissonant emotions, view the sets of positive and negative images used in Studies 1 and 2.

### Method

#### Participants.

Two approximately gender-balanced UK samples were recruited through Prolific for Studies 3 (*N* = 288, 140 women, 146 men, and 2 non-binary; recruited and participated on 18-Jan-2024) and 4 (*N* = 276, 139 women, 132 men, and 5 non-binary; recruited and participated on 15-May-2024). Participants were compensated approximately £6.00 (some variance due to exchange rate fluctuation) for their participation. All participants provided informed consent via the electronic form at the start of the study. In both studies, participants completed two personality questionnaires using Qualtrics, followed immediately by the Emoticon AACT and the VTT, administered online via Pavlovia (https://pavlovia.org/). Both studies were approved by the University of Winnipeg’s Human Research Ethics Board.

#### Materials.

To avoid inattention and fatigue, we selected the SD4 and the ACME from the original questionnaire battery, and administered them along with the Emoticon AACT (Study 1) and VTT. One attention check item was included to ensure data quality (colour = “green”), but no participant failed this check. Each study took approximately 30 minutes to complete.

#### Procedure.

The Emoticon AACT administered in Studies 3 and 4 was identical to Study 1, with the present studies administered online rather than in-lab, and immediately following the questionnaires rather than some months later, as was the case in Studies 1 and 2. During the VTT, participants were shown in a random order the 48 images used in Phase 1 of the AACT, half of which had been previously seen, depending on participants’ prior choices. Participants could press the up or down arrow on their keyboard to increase or decrease the viewing time on each trial. Each image was shown for 4.0 s by default, with each key press adjusting the viewing time by 200 ms. A dynamic timer bar, displayed to the right of the image, emptied faster if participants pressed the down-arrow key and slower if they pressed the up-arrow key, showing the remaining time.

Because participants were paid for their time, the general incentives were aligned in favour of pressing the down key [67], regardless of the nature of the image. To offset this general incentive, participants earned a single point for the first 30 up-presses on a trial. In Study 3, points could be earned during the negative images but not during positive images, and in Study 4, points could be earned during the positive images but not the negative images. This provided an unimpeded sample of wanting (absence of points) during the positive images in Study 3, and an unimpeded sample of wanting during the negative images in Study 4. Points in this task served to increase up key usage rather than to uncover the otherwise hidden preferences of the participants. A running tally of the total points earned throughout the VTT was displayed above the image on each trial. Notice, if the image on the screen was unwanted, the participant might press the down key to remove it in the hopes of seeing more wanted images. The primary measures on each trial were a) the average viewing time and b) the number of up and/or down key presses.

### Results

#### Emoticon approach-avoidance conflict task.

Bivariate correlations between Study 3 predictors (S6 Table in S1 File) and AACT behaviour as measured by switch point and negative approach are shown in Table 4. Participants approached the frowny icon in Phase 1 (Phase 1 negative approach) on 31.8% of trials (*SD* = 0.29), and in Phase 2 (Phase 2 negative approach) on 66.5% of trials (*SD* = 0.29). Mean response latency was greater in Phase 2 compared to Phase 1, *M*_*diff*_ = 0.41, *t*(287) = 2.23, *p* = .022, η^2^ = 0.018. Switch point and Phase 2 negative approach were strongly and inversely correlated, *r*(286) = − .935, *p* < .001. Nearly half of participants (48.6%) exhibited a switch point of 1, with 100 of these 140 participants approaching the frowny icon for 0 point or 1 point, and 40 who approached for 1 but not 0 points. The distinction between approaching for 1 point versus approaching for both 0 and 1 point was not associated with any ACME empathy facet, nor any Dark Tetrad tendencies.

**Table 4:** Personality measure descriptive statistics and bivariate correlations with AACT behaviour in Studies 3 and 4.

Study	Scale	*M*	*SD*	Range	α	AACT		
						P1 NA	P2 NA	SP
Study 3	Gender: M = 1; W = 2					−.013	−.094	.117*
	SD4							
	Machiavellianism	3.32	.616	1.29–5.00	.71	.066	.118*	−.112
	Narcissism	2.50	.755	1.00–4.57	.81	.060	.118*	−.121*
	Psychopathy	1.83	.704	1.00–4.86	.82	.122*	.094	−.098
	Sadism	2.24	.870	1.00–4.86	.84	.096	.144*	−.141*
	ACME							
	Resonance	4.21	.614	2.17–5.00	.61	−.173**	−.165**	.175**
	Cognitive	3.72	.615	1.17–5.00	.91	−.055	.020	−.004
	Dissonance (lack)	4.43	.593	2.23–5.00	.90	−.169**	−.208**	.203**
Study 4	Gender: M = 1; W = 2					−.064	−.143*	.129*
	SD4							
	Machiavellianism	3.34	.573	1.29–5.00	.71	.112	.194**	−.150*
	Narcissism	2.48	.736	1.00–4.71	.83	.074	.224**	−.190**
	Psychopathy	1.85	.692	1.00–4.86	.81	.135*	.145*	−.136*
	Sadism	2.21	.865	1.00–4.86	.85	.073	.158**	−.146*
	ACME							
	Resonance	4.16	.574	2.00–5.00	.86	−.179**	−.115	.126*
	Cognitive	3.65	.618	1.17–4.92	.91	−.038	.143*	−.108
	Dissonance (lack)	4.36	.594	2.33–5.00	.90	−.140*	−.132*	.129*

*Note: n = *288 (Study 3) and 276 (Study 4). AACT = Approach-Avoidance Conflict Task, P1 NA = Phase 1 negative approach; P2 NA = Phase 2 negative approach; SP = switch point; SD4 = Short Dark Tetrad; ACME = Affective and Cognitive Measure of Empathy.

*p < .05, **p < .01.

SD4 traits and ACME facets showed significant associations with AACT behaviour in Study 3 (see Table 4). SD4 sadism was positively correlated with Phase 2 negative approach and negatively correlated with switch point. Unlike Study 1, SD4 narcissism showed the same pattern as SD4 sadism. Negative approach also reached statistical significance for SD4 Machiavellianism. SD4 psychopathy was not significantly associated with either switch point or Phase 2 negative approach, but was positively associated with Phase 1 negative approach. ACME dissonance was associated with a lower Phase 1 negative approach score, a lower Phase 2 negative approach score, and a higher switch point. Like Study 1, a multiple regression with SD4 traits as predictor variables and our two outcome variables (i.e., P2 NA and SP) yielded no significance (Table 5). The same was true when gender was entered as a control variable (S7 Table in S1 File).

**Table 5:** Studies 3 and 4 multiple linear regressions predicting switch point and Phase 2 negative approach from the Dark Tetrad during the AACT.

Study	Variable	*B*	*SE*	β	*Robust* *SE*	*t*	*p*
Study 3	Switch Point						
	SD4 Machiavellianism	−.075	.114	−.044	.125	−0.606	.545
	SD4 Narcissism	−.122	.094	−.087	.092	−1.320	.188
	SD4 Psychopathy	.025	.115	.016	.115	0.215	.830
	SD4 Sadism	−.135	.090	−.111	.087	−1.551	.122
	Phase 2 Negative Approach						
	SD4 Machiavellianism	.024	.031	.051	.033	0.719	.473
	SD4 Narcissism	.032	.026	.084	.025	1.257	.210
	SD4 Psychopathy	−.010	.031	−.025	.028	−.368	.713
	SD4 Sadism	.039	.024	.117	.023	1.657	.099
Study 4	Switch Point						
	SD4 Machiavellianism	−.120	.123	−.067	.131	−.910	.364
	SD4 Narcissism	−.193	.095	−.138	.090	−2.134	.034*
	SD4 Psychopathy	−.038	.114	−.025	.112	−0.339	.735
	SD4 Sadism	−.078	.092	−.066	.101	−0.778	.437
	Phase 2 Negative Approach						
	SD4 Machiavellianism	.054	.034	.107	.038	1.447	.149
	SD4 Narcissism	.064	.027	.163	.025	2.600	.010*
	SD4 Psychopathy	.008	.032	.018	.032	0.245	.806
	SD4 Sadism	.020	.026	.058	.028	0.701	.484

*Note:* Valid *n*’s = 288 (Study 3) and 276 (Study 4), SD4 = Short Dark Tetrad, *t* and *p* on the basis of the robust *SE.*

* *p* < .05.

In Study 4, participants approached the frowny icon in Phase 1 (Phase 1 negative approach) on 34.2% of trials (*SD* = 0.29), and in Phase 2 (Phase 2 negative approach) on 65.9% of trials (*SD* = 0.29). Mean response latency was greater in Phase 2 compared to Phase 1, *M*_*diff*_ = 0.24, *t*(275) = 3.56, *p* < .001, η^2^ = 0.044. Switch point and Phase 2 negative approach were strongly and inversely correlated, *r*(274) = − .947, *p* < .001.

Bivariate correlations between Study 4 predictors (S8 Table in S1 File) and AACT behaviour are shown in Table 4. All four Dark Tetrad traits showed significant positive correlations with Phase 2 negative approach and negative correlations with switch point. ACME dissonance showed negative associations with Phase 1 negative approach and positive associations with switch point. A regression of SD4 traits and gender (S9 Table in S1 File) yielded a unique contribution of narcissism to approach the frowny icon in Phase 2 (P2 NA), *β* = .158, *t*(265) = 2.30, *p* = .022, and a marginally significant unique contribution to switch point, *β* = −.134, *t*(186) = −1.93, *p* = .054.

#### Viewing time task.

Fewer participants completed the VTT than those that completed the AACT due to software incompatibilities (or fatigue), but the samples remained roughly gender balanced. Outliers based on excessive up- or down-key presses were removed on the basis of their individual z-scores because these data could be an artefact of their keyboard being set on “repeat” (+3.0 or above; 8 removed from Study 3; 3 removed from Study 4). These participants were not removed from the AACT task results. As shown in Table 6, up key presses were greater when points were offered, whether for negative images (Study 3) or positive images (Study 4). This resulted in increased viewing times for negative vs. positive images in Study 3, and vice versa in Study 4.

**Table 6:** Per trial descriptive statistics for dependent variables in the viewing-time task.

Variable	Study 3			Study 4		
	*M*	*SD*	Range	*M*	*SD*	Range
View Time						
Positive	3.81**	1.49	1.53–10.57	8.16**	2.34	2.84–15.09
Negative	7.39	2.94	1.52–19.06	2.98	1.15	1.29–8.01
Up Presses						
Positive	4.43**	9.88	0–75.44	29.54**	12.78	0–107.32
Negative	25.37	13.62	0–82.88	1.75	5.66	0–65.36
Down Presses						
Positive	3.73**	3.61	0–14.08	3.42**	4.10	0–17.96
Negative	4.40	4.09	0–15.00	5.53	3.86	0–19.16

*Note:* Study 3 (*n* = 207, men = 105, women = 101, non-binary = 1); Study 4 (*n = *230, men = 109, women = 116, non-binary = 5).

Positive versus negative: *p < .05, **p < .01.

The VTT results for Studies 3 and 4 are shown in Table 7. Most of the VTT variance associated with aversive personality involved responses to positive images rather than negative images. In Study 3, SD4 narcissism and sadism scores were associated with increased down key pressing when positive images were displayed, which had the effect of significantly shortening the viewing time of positive images for participants high in SD4 sadism. Shorter viewing times for positive images were also associated with SD4 psychopathy (Table 7). ACME affective dissonance was associated with longer viewing times and fewer down presses for positive images. For negative images, there were no differences in viewing time as a function of Dark Tetrad tendencies, though SD4 narcissism was associated with more down key presses during negative images, and ACME dissonance was associated with fewer up key presses (Table 7). Among the SD4, multiple regression analyses (S10 Table in S1 File) revealed that sadism significantly predicted down key presses, β = .170, *t*(202) = 2.02, *p* = .044, and viewing time, β = −.213, *t*(202) = −2.55, *p* = .011, for positive images, but not negative images, when controlling for the other Dark Tetrad traits.

**Table 7:** Personality measure bivariate correlations with VTT behaviour in Studies 3 and 4.

Study	Scale	PVT	NVT	UP	UN	DP	DN
Study 3	Gender: M = 1; W = 2	−.213**	-. 114	.160*	−.186**	−.217**	.081
	SD4						
	Machiavellianism	−.136	.120	−.204**	.099	.094	−.018
	Narcissism	−.076	−.013	−.017	.090	.162*	.183**
	Psychopathy	−.155*	.077	−.153*	.128	.130	.026
	Sadism	−.246**	.030	−.157*	.083	.191**	.045
	ACME						
	Resonance	.119	−.108	.117	−.101	−.074	.072
	Cognitive	.056	.047	.069	.080	−.022	.037
	Dissonance (lack)	.215**	−.081	.116	−.193**	−.196**	−.095
Study 4	Gender: M = 1; W = 2	.284**	.033	.101	.020	−.336**	−.033
	SD4						
	Machiavellianism	.048	.160*	.009	.122	.140*	−.085
	Narcissism	−.027	.041	.115	.092	.138*	.008
	Psychopathy	−.008	.081	.021	.079	.165*	.003
	Sadism	−.108	−.028	−.062	−.035	.305**	.049
	ACME						
	Resonance	.191**	−.061	.000	−.035	−.190**	−.027
	Cognitive	.099	−.019	−.031	−.021	−.099	−.031
	Dissonance (lack)	.194**	.011	.050	−.027	−.260**	−.079

*Note:* Study 3 (*n* = 207); Study 4 (*n* = 230). VTT = Viewing-Time Task; PVT = positive image viewing time; NVT = negative image viewing time; UP = up-presses for positive images; UN = up-presses for negative images; DP = down-presses for positive images; DN = down-presses for negative images; SD4 = Short Dark Tetrad; ACME = Affective and Cognitive Measure of Empathy. *p < .05, **p < .01.

In Study 4, increased down key presses during positive images were associated with all Dark Tetrad traits, especially SD4 sadism (Table 7). As was the case in Study 3, low scores on the ACME affective dissonance scale were associated with fewer down key presses when positive images were displayed, with ACME affective resonance also showing significance. The only significant association for negative images was a positive correlation between SD4 Machiavellianism and viewing time (Table 7). Multiple regression analyses (S11 Table in S1 File) revealed similar results as Study 3, with sadism predicting down key presses, β = .312, *t*(225) = 3.73, *p* < .001, for positive images, but not negative images, when controlling for the other Dark Tetrad traits.

### Discussion

The associations between Dark Tetrad traits and AACT choices in Studies 3 and 4 were broadly consistent with the patterns observed in Study 1 with the exception of narcissism. In both Studies 3 and 4, sadism, Machiavellianism, and narcissism were associated with increased approach toward negative images, with fewer points required to motivate this behaviour. In Study 4, psychopathy also showed significant associations along these lines. These findings are consistent with Study 1 in which it was found that the Emoticon AACT draws out dark tendencies more broadly, perhaps on the basis of high BAS activation and low BIS activation. Of note, SD4 narcissism was significantly associated with switch point and negative approach in Studies 3 and 4, when no significance was found in Study 1. This association is consistent with the understanding of grandiose narcissism as a high approach/low avoidance trait [39], and supports our original hypothesis. The variation in narcissism significance across studies could be a consequence of sampling error or the unbalanced sample used in Study 1. We additionally found no unique Dark Tetrad predictors of switch point in our multiple regression analyses, supporting our conclusion that the Emoticon AACT taps into broad high-approach/low-avoidance tendencies among those with malevolent traits, rather than teasing them apart. The ACME facets of affective resonance and affective dissonance also showed significant associations with approach behaviour in both phases of Studies 3 and 4, consistent with our earlier findings.

Rather than predicting increased viewing times and up presses for negatively valenced images, the Dark Tetrad traits were associated with decreased viewing times and increased down presses for positively valenced images. Our regression analyses were able to tease apart sadistic tendencies from the rest of the Dark Tetrad on trials with positive images. Everyday sadists feel a mixture of negative and positive emotions when viewing images most people would label positive [2], and greater negative emotions in response to positive video clips [3]. Our VTT data suggest that dislike for positive images pushed the participants towards the frowny icon during the Emoticon AACT. Whatever attraction the negative images held for those with sadistic tendencies, the pulling effect, was relatively less important.

## General discussion

Socially aversive personality traits played a role in the observed tendencies of participants to choose to approach aversive stimuli in a computer task, especially when points were available. In Study 1, we used a modified version of an Emoticon AACT [42] with negative or positive images presented contingently on the participants’ choices to approach a frowny face or a smiley face icon, respectively. Fewer points were required to lure Machiavellians to choose the frowny icon, with the tactics subscale of the TDMS predicting their choices. Psychopathy facets of boldness and meanness were also correlated with a decision to approach the frowny face, which was corroborated by our switch point measure. The switch point data also indicated that sadism and affective empathy (both resonance and dissonance scales) were significant factors in Emoticon AACT behaviour. With the positive and negative images on screen when the participants were making their decisions in the Image AACT in Study 2, vicarious sadism and affective dissonance were significant correlates of both Phase 2 negative approach and switch point. Using gender balanced samples, Studies 3 and 4 examined whether the choices of participants with high levels of Dark Tetrad traits were due to characteristics of the negative or positive images used in Study 1. The results of a viewing-time task [65] conducted after the Emoticon AACT suggested that sadists would shorten their viewing time of the positive images but not lengthen their viewing time of negative images. These data provide additional evidence in a behavioural task that people high in sadism feel negative emotions when looking at peaceful or happy images [2,3], which acted as an additional source of motivation for choosing to approach the frowny icon in the Emoticon AACT.

Why did people with sadistic and dissonant emotional traits find the positive slides so unappealing? About one-third of the images depicted positive situations with people (e.g., two smiling girls camping with their father, mother pushing a stroller, and a happy boy in a swimming pool, a person reading a book while resting a hammock strung between palm trees), whereas about another one-third of the images depicted peaceful scenes in the absence of people (red kayaks on a dock, mountains, sunsets, and beaches). Evidence that people high in sadism generally dislike these kinds of positive images was found in Studies 3 and 4 on all measures, including less viewing time, more down-key presses, and fewer-up key presses. To some extent, the patterns found with sadism were shared by the other Dark Tetrad traits. Although sadism was the trait most predictive of negative responses to the positive images, psychopathy (less viewing time, fewer up presses), narcissism (more down presses), and Machiavellianism (fewer up presses) showed significant associations with some dependent variables in the viewing time task.

The negative images were not specifically selected to appeal to sadistic tastes, although there was a violent photo of a woman getting her hair pulled. One of the items on the SD4 sadism subscale is “I enjoy watching violent sports”. There were no sports violence photos in this set of negative images. Our negative images were broadly aversive, depicting graphic scenes of pain and human suffering, but also included property destruction, rotten food, dirty toilets, and relatively benign images of garbage. This might explain the relatively weak sadistic attraction to negative images in the present studies. On the other hand, sadism predicts reduced defensive reactions to aversive noises [68] and a preference for bitter tastes that others may find aversive [69], suggesting a broader influence on responses to negative stimuli, not just those involving suffering. Future work might examine the Dark Tetrad in relation to approach and avoidance of positive and negative stimuli of various modes (e.g., images, sounds, smells, tastes, etc.) to obtain a complete picture of these tendencies.

Beyond the Dark Tetrad traits, ACME affective dissonance was consistently associated with AACT performance across Studies 1–4. Empathy typically refers to feeling a similar emotion as another person is feeling (affective resonance empathy) and understanding what emotion another person is feeling (cognitive empathy). Affective dissonance describes the trait of feeling opposite or differently than another person, which is an emotional experience that is incompatible with affective empathy [4]. This construct encompasses sadism but casts a wider net to include displeasure at the positive emotions of others (e.g., “When my friends are having a good time I often get angry” and “people who are cheery disgust me”). Consistent with a pattern of socially-discordant emotions, participants high in sadism or affective dissonance likely found negative images to be appealing, and positive images to be unappealing, which may explain the observed associations with AACT and VTT behaviour.

Evidence that a conflict was induced can be inferred from the significant increase in reaction time from Phase 1 to Phase 2. The magnitude of the difference was greatest in the Image AACT in Study 2, but was also evident with the Icon AACT (with the exception of Study 1). This pattern most likely reflects the increased cognitive processing demands associated with conflict decision making. Because they must first weigh the potential costs and benefits of their choice, participants often took longer to make a decision in Phase 2 of the AACT. Previous research suggests the anterior cingulate cortex (ACC) plays a central role in decision making when cognitive control is allocated through a cost/benefit analysis [70], and this is reflected by increased activation of the ACC when individuals make approach-avoidance conflict decisions [71]. For example, Abivardi et al. [72] explored the neural correlates of conflict decision making by asking participants to collect tokens while at risk of being captured by a virtual predator. They observed a blood-oxygen-level-dependent (BOLD) signal increase in the anterior hippocampus during avoidance behaviour, but also found a BOLD signal increase in the ACC, indicating participants were experiencing cognitive conflict. Another neuroimaging study [46] of individuals with MDD identified an association between ACC activation and avoidance behaviour. They reported that MDD was associated with reduced avoidance-related ACC activation. MDD was also related to reduced reward sensitivity, suggesting reward sensitivity and ACC activation could be linked [73]. Further, if ACC activation is a reliable indicator of avoidance behaviour in AACTs [46], there is a possibility that people high in sadistic traits may exhibit less conflict and less ACC activation in this type of task, something that future studies could explore [42,44].

Taken together, the results of our four studies provide valuable insight into the dispositional influences on behaviour in approach-avoidance conflict. Most past research has used the AACT to gain insight into maladaptive behaviours in clinical disorders like anxiety disorders [42,44]. In contrast, our study focused on subclinical traits with subclinical samples. Our results align with previous AACT findings linking dispositional anger with greater approach behaviour when provoked or frustrated [74]. It seems that people high in the Dark Tetrad traits, dispositional anger, and affective dissonance are undeterred by aversive experiences. As all of these traits are associated with aggressive behaviour [4,5,75], future research might examine AACT behaviour as a mechanism of those relationships.

Our findings may also have practical implications for organizational settings. Specifically, the AACT patterns highlight a surprising character strength of people with Dark Tetrad traits in the subclinical range. When properly incentivized, these individuals will forgo normatively pleasant experiences in favor of normatively aversive experiences. Such tendencies would be beneficial in occupations that require individuals to fearlessly advance toward danger. In support of this view, first responders have bold and fearless traits that facilitate approach toward aversive stimuli (e.g., fires, perpetrators, injured people, etc.) [76]. Other work links appetitive aggression to lower post-traumatic-stress symptoms among soldiers [77], which suggests that negative approach motivation is advantageous in violent combat. Given these patterns, AACT behaviour might be a useful addition to employee selection processes for roles that require courage under threat.

### Limitations

Some limitations derive from the AACT and VTT themselves as implemented in the present studies. The AACT requires the participant to imagine themselves in the situation, and then press a computer key. Whether they did so might depend on their personality traits. The AACT is designed to tap into the decision to move, not the movement itself. Participants never experienced the perception of moving in space, which some other approach-avoidance tasks emphasize [78]. With the advent of mobile electroencephalography [79], future studies might examine the neural activity correlated with a) the decision to move, b) the perception of movement, and c) the movement itself. A neutral category of images that was selectively associated with points in the VTT might have permitted us to directly compare the positive and negative images.

There are a number of differences between the Emoticon AACT used in Studies 1, 3, and 4 and the Image AACT used in Study 2. We have emphasized the role of anticipatory anxiety. Not knowing what negative image lay behind the choice of the frowny icon in the Emoticon AACT presumably evoked physical anxiety in Study 1. It should be acknowledged that other differences, no matter how trivial, might be responsible for the differences found between Studies 1 and 2. Further work is needed to characterize the role of aversive personality in the Image AACT. Desensitization toward graphic images as these types of scenes have become more common in everyday life [80], which may have impacted the results found in the Image AACT.

Additional methodological limitations should also be noted. First, although the use of Prolific in Studies 3 and 4 permitted us to collect data from gender-balanced samples, the shift to an online study came with the loss of experimental control. Some participants may have completed the tasks in a public environment with the potential of being observed by others. If so, the presence of other people may have influenced their behaviour in the AACT or VTT tasks. Future studies could address this limitation by asking participants if they believed they were being watched or were concerned about privacy during the task. Environmental distractions may also have been an issue, but the attention-check item ensured an adequate level of attention during the tasks. Second, all studies used convenience samples that may not be representative of the general population (e.g., due to self-selection bias). Psychology students and Prolific workers may differ in important ways from the general public. However, the similar pattern of results between student and Prolific samples provides evidence that the findings can be generalized more broadly. Third, the AACT and VTT are artificial computer tasks, and the results may not generalize to situations encountered in everyday life. For instance, the psychological factors involved in moving a manikin figure toward a negative image on a computer screen may not be the same as those involved in physically approaching a dangerous object and putting oneself at risk. These limitations could be addressed in future studies by examining responses to approach-avoidance conflicts in naturalistic settings with diverse samples.

## Conclusion

Perhaps the most striking feature of sadism is a willingness to pay a high price to harm others [1]. Less is known about how people high in sadism and other socially aversive traits behave in more positive situations in everyday life. Our data reveal that highly sadistic individuals will press a down key to reduce their viewing time of peaceful landscapes or happy people, which is a relatively small price to pay, especially when it lessens the time required to complete a paid task. Whether sadists will pay an even higher cost to avoid what others perceive as positive situations remains to be determined.

## Supporting information

S1 File All supporting information. (DOCX)
