# Supplementary material for: Examining motivational profiles in the dark personality tetrad using an approach-avoidance conflict task
Source: PLoS One. 2025 Jul 29;20(7):e0327609. doi: 10.1371/journal.pone.0327609 (PMC12306764; doi:10.1371/journal.pone.0327609)
Supplement: S1 File — (DOCX) [file pone.0327609.s001.docx]

**Supporting Information: Table of Contents**

Data are available for download on the Open Science Framework ([https://osf.io/gchnm](https://osf.io/wh6zr)).

PsychoPy experiment files are available for download at <https://doi.org/10.5281/zenodo.14532285>.

**S1A Table. Images.**

**S1B Supporting Information. Task Instructions.**

**S1C Supporting Information. PsychoPy Links for Studies 1, 2, and 3.**

**S2 Table. Study 1 Intercorrelations.**

**S3 Table. Study 1 AACT Multiple Regressions.**

**S4 Table. Study 2 Intercorrelations.**

**S5 Table. Study 2 AACT Multiple Regressions.**

**S6 Table. Study 3 Intercorrelations.**

**S7 Table. Study 3 AACT Multiple Regressions.**

**S8 Table. Study 4 Intercorrelations.**

**S9 Table. Study 4 AACT Multiple Regressions.**

**S10 Table. Study 3 VTT Multiple Regressions.**

**S11 Table. Study 4 VTT Multiple Regressions.**

**Table S1A**

*Images*

| *Image Type* | *Source* | *File* | *Image Type* | *Source* | *File* |
| --- | --- | --- | --- | --- | --- |
| Positive | DIRTI | 1094_animals neutral | Negative | DIRTI | 1015_food |
| Positive | DIRTI | 1142_body products neutral | Negative | DIRTI | 1156_injuries_infections |
| Positive | OASIS | Barbeque 2 | Negative | DIRTI | 1176_injuries_infections |
| Positive | OASIS | Beach 1 | Negative | DIRTI | 1213_death |
| Positive | OASIS | Camping 1 | Negative | DIRTI | 1260_hygiene |
| Positive | OASIS | Car race 2 | Negative | OASIS | Angry face 3 |
| Positive | OASIS | Cat 2 | Negative | OASIS | Angry face 4 |
| Positive | OASIS | Dessert 8 | Negative | NAPS | Animals_074_h |
| Positive | OASIS | Eating 3 | Negative | NAPS | Animals_077_h |
| Positive | Madrid | EM0125 | Negative | OASIS | Bored pose 4 |
| Positive | Madrid | EM0147 | Negative | OASIS | Car accident 1 |
| Positive | Madrid | EM0208 | Negative | OASIS | Depressed pose 3 |
| Positive | Madrid | EM0270 | Negative | OASIS | Destruction 8 |
| Positive | Madrid | EM0271 | Negative | OASIS | Dirt 1 |
| Positive | Madrid | EM0299 | Negative | Madrid | EM0326 |
| Positive | Madrid | EM0300 | Negative | Madrid | EM0327 |
| Positive | Madrid | EM0307 | Negative | Madrid | EM0331 |
| Positive | Madrid | EM0413 | Negative | Madrid | EM0391 |
| Positive | Madrid | EM0422 | Negative | Madrid | EM0484 |
| Positive | Madrid | EM0438 | Negative | Madrid | EM0563 |
| Positive | Madrid | EM0446 | Negative | Madrid | EM0596 |
| Positive | Madrid | EM0533 | Negative | Madrid | EM0613 |
| Positive | Madrid | EM0544 | Negative | Madrid | EM0618 |
| Positive | Madrid | EM0660 | Negative | Madrid | EM0692 |
| Positive | Madrid | EM0699 | Negative | Madrid | EM0710 |
| Positive | Madrid | EM0702 | Negative | Madrid | EM0722 |
| Positive | NAPS | Faces_078_h | Negative | NAPS | Faces_009_h |
| Positive | NAPS | Faces_091_v | Negative | NAPS | Faces_032_h |
| Positive | NAPS | Faces_111_h | Negative | NAPS | Faces_283_h |
| Positive | NAPS | Faces_137_h | Negative | NAPS | Faces_284_h |
| Positive | NAPS | Faces_225_v | Negative | NAPS | Faces_293_h |
| Positive | NAPS | Faces_235_v | Negative | NAPS | Faces_362_v |
| Positive | NAPS | Faces_315_h | Negative | NAPS | Faces_365_v |
| Positive | NAPS | Faces_333_h | Negative | NAPS | Faces_367_h |
| Positive | NAPS | Faces_334_h | Negative | OASIS | Fence 4 |
| Positive | OASIS | Fireworks 6 | Negative | OASIS | Frustrated pose 2 |
| Positive | OASIS | Fireworks 7 | Negative | OASIS | Frustrated pose 5 |
| Positive | OASIS | Flowers 8 | Negative | OASIS | Funeral 1 |
| Positive | OASIS | Lake 9 | Negative | OASIS | Gun 5 |
| Positive | OASIS | Lake 10 | Negative | OASIS | Injury 1 |
| Positive | NAPS | Landscapes_140_v | Negative | NAPS | People_038_h |
| Positive | NAPS | Landscapes_180_h | Negative | NAPS | People_237_h |
| Positive | OASIS | Mother 5 | Negative | NAPS | People_239_h |
| Positive | OASIS | Musician 1 | Negative | OASIS | Police 5 |
| Positive | NAPS | Objects_077_h | Negative | OASIS | Sad pose 1 |
| Positive | NAPS | Objects_086_h | Negative | OASIS | Scary face 2 |
| Positive | NAPS | People_190_h | Negative | OASIS | Shark 4 |

*Note*. DIRTI: Disgust-RelaTed-Images. Madrid: EmoMadrid. NAPS: Nencki Affective Picture System. OASIS: Open Affective Standardized Image Set.

**Supporting Information S1B. Task Instructions.**

Instructions for the Emoticon AACT used in Study 1 (square brackets indicate the alternative instructions for the Image AACT in Study 2):

Welcome to Manikin. Please make sure you are seated comfortably, can see the screen, and can reach the keyboard.

On each trial, look at the fixation dot in the center of the screen.

You may opt out of continuing the task at any point, and there is no penalty for doing so (simply press ESC).

There are two parts to this study, phase 1 and phase 2.

When you are ready, press SPACEBAR to advance to the first phase.

In the first part of this experiment, you will be presented with two images on the screen, one on the left, the other on the right.

One of the images will be a smiley face [positive], the other will be a frowny face [negative].

Be alert, the smiley and frowny faces [POSITIVE image] may appear on different sides of the screen.

You will also see a stick figure manikin in the centre of the screen, which represents YOU.

Your task is to walk the manikin (the stick-figure version of you) toward either the SMILEY or FROWNY face, which will reveal either a positive image for the smiley face or negative image for the frowny face [toward the POSITIVE image, putting increased distance between you and the negative image]

Press the ‘right’ or ‘left’ arrow key on the keyboard to move the manikin in that direction.

Your reaction time and choices [accuracy] are being recorded, so please answer as quickly as you can without making any mistakes.

Press SPACEBAR to begin phase 1.

In the second part of this experiment you will be given the opportunity to earn points if you approach the frowny face [negative] image.

The point value available for approaching a frowny face will appear above it [above that image].

The amount of points up for grabs on each trial varies (0, 1, 5, 10, 25, or 50 points).

If you walk toward the smiley face [positive image], you will continue to earn smiles. Walk toward the frowny face [negative image] and you potentially rewarded with points.

There are no right or wrong answers.

Press SPACEBAR to begin phase 2.

Instructions for the Emoticon AACT and viewing time tasks in Study 3:

Welcome to Manikin.

Please make sure you are seated comfortably, can see the screen, and can reach the keyboard.

There are three parts to this study, Phase 1, Phase 2, and Phase 3.

On each trial, look at the fixation dot in the centre of the screen.

You may opt out of continuing the task at any point, and there is no penalty for doing so (simply press ESC).

When you are ready, press SPACEBAR to advance to Phase 1.

In Phase 1, you will be presented with two icons on the screen, one on the left, the other on the right.

One of the images will be a smiley face, the other will be a frowny face.

Be alert, the smiley and frowny faces may appear on different sides of the screen.

You will also see a stick figure manikin in the center of the screen, which represents YOU.

Your task is to walk the manikin (stick figure version of you) toward either the SMILEY or FROWNY face, which will reveal either a positive image for the smiley face, or negative image for the frowny face.

Press the ‘right’ or ‘left’ arrow keys on the keyboard to move the manikin in that direction.

Your reaction time and choices are being recorded, so please answer as quickly as you can without making any mistakes.

Press SPACEBAR to begin Phase 1.

In Phase 2, you will have the opportunity to earn points if you approach the frowny face.

The point value available for approaching a frown face will appear above it.

The amount of points available varies on each trial (0, 1, 5, 10, 25, or 50 points).

If you walk toward the smiley face, you will continue to earn positive images. Walk toward the frowny face, and you are potentially rewarded with points.

There are no right or wrong answers.

Press SPACEBAR to begin Phase 2.

In Phase 3, you will be presented with images you might have seen in Phase 1,

To the right of the image, a timer bar will begin decreasing, indicating how much time is left. The default viewing time is 4 seconds.

Pressing the ‘up’ arrow on the keyboard repeatedly will cause the timer bat to fill up, while pressing the ‘down’ arrow repeatedly will cause it to empty faster.

When the timer bar runs out, you will be shown the next image.

Press SPACEBAR to continue to the rest of the Phase 3 instructions.

If a negative image is displayed on the screen, pressing the ‘up’ arrow will earn you 1 point per press, in addition to adding viewing time.

If a positive image is displayed, pressing the ‘up’ arrow will add viewing time, but will NOT earn you any points.

Your cumulative point total will be displayed above the image.

You may view each image for as long as you wish, but you will not be able to earn more than 30 points per negative image.

Please respond using the index finger of your dominant hand. There are 48 images in this phase, with a 0,5 second separation between images.

When you are ready, press the SPACEBAR to begin Phase 3.

**Supporting Information S1C. PsychoPy Links for Studies 1, 2, and 3.**

Study 1:https://run.pavlovia.org/publicprojects/emoticonmanikindemoexp1

Study 2:https://run.pavlovia.org/publicprojects/imagemanikindemoexp2

Study 3:https://run.pavlovia.org/publicprojects/emoticonmanikinvttdemoexp3

**Table S2**

*Study 1 Intercorrelations*

|  | M | SD | Gender | 1 | 2 | 3 | 4 | 5 | 6 | 7 | 8 | 9 | 10 | 11 | 12 | 13 | 14 | 15 | 16 | 17 | 18 | 19 | 20 | 21 | 22 | 23 | 24 | 25 |
| --- | --- | --- | --- | --- | --- | --- | --- | --- | --- | --- | --- | --- | --- | --- | --- | --- | --- | --- | --- | --- | --- | --- | --- | --- | --- | --- | --- | --- |
| SD4 |  |  |  |  |  |  |  |  |  |  |  |  |  |  |  |  |  |  |  |  |  |  |  |  |  |  |  |  |
| 1. Machiavellianism | 3.47 | .716 | -0.199* | (.78) |  |  |  |  |  |  |  |  |  |  |  |  |  |  |  |  |  |  |  |  |  |  |  |  |
| 1. Narcissism | 2.97 | .721 | 0.000 | 0.104 | (.76) |  |  |  |  |  |  |  |  |  |  |  |  |  |  |  |  |  |  |  |  |  |  |  |
| 1. Psychopathy | 1.87 | .748 | -0.186* | 0.002 | 0.220* | (.83) |  |  |  |  |  |  |  |  |  |  |  |  |  |  |  |  |  |  |  |  |  |  |
| 1. Sadism | 2.37 | .912 | -0.329** | 0.325** | 0.026 | 0.402** | (.83) |  |  |  |  |  |  |  |  |  |  |  |  |  |  |  |  |  |  |  |  |  |
| TDMS |  |  |  |  |  |  |  |  |  |  |  |  |  |  |  |  |  |  |  |  |  |  |  |  |  |  |  |  |
| 1. Views | 3.85 | .969 | -0.097 | 0.373** | -0.041 | 0.194* | 0.328** | (.69) |  |  |  |  |  |  |  |  |  |  |  |  |  |  |  |  |  |  |  |  |
| 1. Tactics | 3.22 | 1.152 | -0.217* | 0.332** | -0.140 | 0.145* | 0.417** | 0.368** | (.79) |  |  |  |  |  |  |  |  |  |  |  |  |  |  |  |  |  |  |  |
| B-PNI |  |  |  |  |  |  |  |  |  |  |  |  |  |  |  |  |  |  |  |  |  |  |  |  |  |  |  |  |
| 1. Grandiose | 3.69 | .872 | -0.087 | 0.516** | 0.306** | 0.114 | 0.319** | 0.137 | 0.155* | (.83) |  |  |  |  |  |  |  |  |  |  |  |  |  |  |  |  |  |  |
| 1. Vulnerable | 3.30 | .951 | 0.093 | 0.376** | -0.006 | 0.196* | 0.240** | 0.263** | 0.258** | 0.611** | (.88) |  |  |  |  |  |  |  |  |  |  |  |  |  |  |  |  |  |
| TriPM |  |  |  |  |  |  |  |  |  |  |  |  |  |  |  |  |  |  |  |  |  |  |  |  |  |  |  |  |
| 1. Boldness | 2.40 | .408 | -0.142* | -0.074 | 0.534** | 0.132 | -0.028 | -0.087 | -0.103 | 0.047 | -0.312** | (.77) |  |  |  |  |  |  |  |  |  |  |  |  |  |  |  |  |
| 1. Meanness | 1.72 | .440 | -0.225* | 0.216* | -0.005 | 0.473** | 0.576** | 0.354** | 0.465** | 0.200* | 0.269** | 0.044 | (.85) |  |  |  |  |  |  |  |  |  |  |  |  |  |  |  |
| 1. Disinhibition | 1.94 | .360 | -0.210* | 0.125 | -0.044 | 0.543** | 0.392** | 0.261** | 0.205* | 0.135 | 0.339** | -0.178* | 0.526** | (.74) |  |  |  |  |  |  |  |  |  |  |  |  |  |  |
| CAST |  |  |  |  |  |  |  |  |  |  |  |  |  |  |  |  |  |  |  |  |  |  |  |  |  |  |  |  |
| 1. Sadism (total) | 1.72 | .666 | -0.314** | 0.151* | 0.030 | 0.364** | 0.718** | 0.182* | 0.321** | 0.232** | 0.209* | 0.032 | 0.660** | 0.394** | (.87) |  |  |  |  |  |  |  |  |  |  |  |  |  |
| 1. Direct Verbal | 2.02 | .951 | -0.201* | 0.099 | -0.017 | 0.212* | 0.514** | 0.075 | 0.250** | 0.210* | 0.256** | -0.033 | 0.531** | 0.339** | 0.813** | (.82) |  |  |  |  |  |  |  |  |  |  |  |  |
| 1. Direct Physical | 1.28 | .552 | -0.143* | 0.072 | 0.137 | 0.356** | 0.477** | 0.194* | 0.275** | 0.197* | 0.147* | 0.118 | 0.556** | 0.362** | 0.747** | 0.451** | (.83) |  |  |  |  |  |  |  |  |  |  |  |
| 1. Vicarious | 1.86 | .968 | -0.369** | 0.172* | 0.001 | 0.341** | 0.703** | 0.192* | 0.262** | 0.160* | 0.098 | 0.032 | 0.526** | 0.276** | 0.840** | 0.439** | 0.530** | (.83) |  |  |  |  |  |  |  |  |  |  |
| ACME |  |  |  |  |  |  |  |  |  |  |  |  |  |  |  |  |  |  |  |  |  |  |  |  |  |  |  |  |
| 1. Affective Resonance | 4.12 | .558 | 0.109 | -0.089 | 0.048 | -0.363** | -0.315** | -0.271** | -0.384** | 0.057 | -0.085 | 0.034 | -0.697** | -0.336** | -0.393** | -0.330** | -0.346** | -0.290** | (.80) |  |  |  |  |  |  |  |  |  |
| 1. Cognitive Empathy | 3.65 | .669 | 0.026 | 0.155* | 0.227** | -0.084 | -0.035 | -0.023 | -0.064 | 0.404** | 0.045 | 0.288** | -0.203* | -0.198* | -0.118 | -0.087 | -0.080 | -0.113 | 0.320** | (.89) |  |  |  |  |  |  |  |  |
| 1. Affective Dissonance | 4.14 | .658 | 0.229** | -0.225* | -0.071 | -0.410** | -0.603** | -0.251** | -0.400** | -0.265** | -0.324** | 0.038 | -0.720** | -0.456** | -0.712** | -0.634** | -0.581** | -0.516** | 0.618** | 0.129 | (.78) |  |  |  |  |  |  |  |
| ASI-3 |  |  |  |  |  |  |  |  |  |  |  |  |  |  |  |  |  |  |  |  |  |  |  |  |  |  |  |  |
| 1. Cognitive Anxiety | 2.60 | 1.006 | 0.042 | 0.040 | -0.012 | 0.083 | -0.074 | 0.072 | 0.013 | 0.155* | 0.334** | -0.269** | 0.014 | 0.201* | -0.092 | -0.077 | 0.027 | -0.129 | -0.087 | -0.016 | -0.079 | (.86) |  |  |  |  |  |  |
| 1. Social Anxiety | 2.93 | .823 | 0.131 | 0.084 | -0.032 | 0.057 | -0.002 | 0.082 | 0.117 | 0.265** | 0.486** | -0.313** | 0.099 | 0.158 | 0.026 | 0.081 | 0.057 | -0.058 | -0.107 | 0.053 | -0.138 | 0.583** | (.70) |  |  |  |  |  |
| 1. Physical Anxiety | 2.48 | .956 | 0.137 | -0.081 | 0.018 | 0.101 | -0.068 | -0.086 | -0.105 | 0.153* | 0.248** | -0.220* | 0.036 | 0.142* | 0.026 | 0.022 | 0.045 | 0.006 | -0.017 | -0.019 | -0.077 | 0.585** | 0.526** | (.84) |  |  |  |  |
| BIS/BAS |  |  |  |  |  |  |  |  |  |  |  |  |  |  |  |  |  |  |  |  |  |  |  |  |  |  |  |  |
| 1. BAS Drive | 2.71 | .606 | -0.230** | 0.146* | 0.208* | 0.247** | 0.119 | -0.025 | 0.062 | 0.203* | 0.136 | 0.248** | 0.209* | 0.087 | 0.127 | 0.017 | 0.182* | 0.141* | -0.118 | 0.005 | -0.111 | -0.013 | 0.039 | 0.050 | (.76) |  |  |  |
| 1. BAS Fun Seeking | 3.06 | .526 | -0.056 | 0.150* | 0.106 | 0.210* | 0.141* | 0.002 | -0.015 | 0.204** | 0.124 | 0.227** | 0.098 | 0.205* | 0.095 | 0.046 | 0.081 | 0.104 | 0.006 | 0.134 | -0.079 | -0.058 | -0.055 | -0.024 | 0.476** | (.60) |  |  |
| 1. BAS Reward | 3.48 | .436 | 0.088 | 0.190* | 0.114 | -0.139 | 0.021 | -0.133 | -0.193* | 0.275** | 0.155* | -0.042 | -0.204* | -0.156* | -0.051 | -0.026 | -0.079 | -0.034 | 0.277** | 0.114 | 0.184* | 0.028 | 0.095 | 0.039 | 0.370** | 0.481** | (.69) |  |
| 1. BIS | 3.21 | .472 | 0.263** | 0.113 | -0.198* | -0.252** | -0.041 | 0.018 | -0.119 | 0.210* | 0.413** | -0.546** | -0.268** | 0.022 | -0.123 | -0.010 | -0.161* | -0.151* | 0.275** | 0.013 | 0.068 | 0.340** | 0.369** | 0.275** | -0.163* | 0.025 | 0.330** | (.71) |

*Note: n* = 196. SD4 = Short Dark Tetrad. TDMS = Two-Dimensional Machiavellianism Scale. B-PNI = Brief Pathological Narcissism Scale. TriPM = Triarchic Psychopathy Measure. CAST = Comprehensive Assessment of Sadistic Tendencies. ACME = Affective and Cognitive Measures of Empathy. ASI-3 = Anxiety Sensitivity Index. BIS/BAS = Behavioural Inhibition System/Behavioural Activation (Approach) System. Gender: man = 1, woman = 2. () = Cronbach’s alpha.

**p* < .05 ***p* < .001

**Table S3**

*Study 1 Multiple Regressions Predicting AACT Switch Point and Phase 2 Negative Approach from the Dark Tetrad (Model 1) and the Dark Tetrad and Gender (Model 2)*

| Model | Variable | *B* | *SE* | β | *t* | *p* |
| --- | --- | --- | --- | --- | --- | --- |
| Switch Point | | | | | | |
| 1 | SD4 Machiavellianism | -.169 | .122 | -.108 | -1.391 | .166 |
|  | SD4 Narcissism | -.074 | .115 | -.048 | -0.639 | .523 |
|  | SD4 Psychopathy | -.011 | .122 | -.007 | -0.086 | .931 |
|  | SD4 Sadism | -.072 | .103 | -.059 | -0.702 | .483 |
| 2 | SD4 Machiavellianism | -.177 | .122 | -.113 | -1.449 | .149 |
|  | SD4 Narcissism | -.063 | .115 | -.041 | -0.546 | .586 |
|  | SD4 Psychopathy | -.019 | .122 | -.013 | -0.157 | .875 |
|  | SD4 Sadism | -.052 | .106 | -.043 | -0.497 | .620 |
|  | Gender (1 = Man, 2 = Woman) | .180 | .210 | .066 | 0.855 | .394 |
| Phase 2 Negative Approach | | | | | | |
| 1 | SD4 Machiavellianism | .041 | .031 | .101 | 1.312 | .191 |
|  | SD4 Narcissism | .005 | .029 | .013 | 0.176 | .861 |
|  | SD4 Psychopathy | .017 | .031 | .044 | 0.539 | .590 |
|  | SD4 Sadism | .024 | .026 | .076 | 0.902 | .368 |
| 2 | SD4 Machiavellianism | .044 | .031 | .109 | 1.397 | .164 |
|  | SD4 Narcissism | .002 | .029 | .006 | 0.078 | .938 |
|  | SD4 Psychopathy | .020 | .031 | .052 | 0.627 | .531 |
|  | SD4 Sadism | .019 | .027 | .061 | 0.708 | .480 |
|  | Gender (1 = Man, 2 = Woman) | -.043 | .054 | -.062 | -0.812 | .418 |

*Note*. Valid *n*’s 192 – 194.

**Table S4**

*Study 2 Intercorrelations*

|  | M | SD | Gender | 1 | 2 | 3 | 4 | 5 | 6 | 7 | 8 | 9 | 10 | 11 | 12 | 13 | 14 | 15 | 16 | 17 | 18 | 19 | 20 | 21 | 22 | 23 | 24 | 25 |
| --- | --- | --- | --- | --- | --- | --- | --- | --- | --- | --- | --- | --- | --- | --- | --- | --- | --- | --- | --- | --- | --- | --- | --- | --- | --- | --- | --- | --- |
| SD4 |  |  |  |  |  |  |  |  |  |  |  |  |  |  |  |  |  |  |  |  |  |  |  |  |  |  |  |  |
| 1. Machiavellianism | 3.43 | .632 | -0.151* | (.72) |  |  |  |  |  |  |  |  |  |  |  |  |  |  |  |  |  |  |  |  |  |  |  |  |
| 1. Narcissism | 2.80 | .753 | -0.073 | 0.210* | (.82) |  |  |  |  |  |  |  |  |  |  |  |  |  |  |  |  |  |  |  |  |  |  |  |
| 1. Psychopathy | 1.86 | .712 | -0.056 | 0.147* | 0.331** | (.80) |  |  |  |  |  |  |  |  |  |  |  |  |  |  |  |  |  |  |  |  |  |  |
| 1. Sadism | 2.34 | .888 | -0.238** | 0.257** | 0.242** | 0.439** | (.82) |  |  |  |  |  |  |  |  |  |  |  |  |  |  |  |  |  |  |  |  |  |
| TDMS |  |  |  |  |  |  |  |  |  |  |  |  |  |  |  |  |  |  |  |  |  |  |  |  |  |  |  |  |
| 1. Views | 3.77 | .947 | 0.022 | 0.181* | -0.034 | 0.218** | 0.155* | (.69) |  |  |  |  |  |  |  |  |  |  |  |  |  |  |  |  |  |  |  |  |
| 1. Tactics | 3.30 | 1.096 | -0.059 | 0.284** | 0.251** | 0.273** | 0.334** | 0.257** | (.75) |  |  |  |  |  |  |  |  |  |  |  |  |  |  |  |  |  |  |  |
| B-PNI |  |  |  |  |  |  |  |  |  |  |  |  |  |  |  |  |  |  |  |  |  |  |  |  |  |  |  |  |
| 1. Grandiose | 2.58 | .854 | -0.089 | 0.439** | 0.382** | 0.177* | 0.268** | 0.046 | 0.208* | (.84) |  |  |  |  |  |  |  |  |  |  |  |  |  |  |  |  |  |  |
| 1. Vulnerable | 2.16 | .886 | 0.123 | 0.217* | 0.039 | 0.104 | 0.180* | 0.105 | 0.103 | 0.584** | (.87) |  |  |  |  |  |  |  |  |  |  |  |  |  |  |  |  |  |
| TriPM |  |  |  |  |  |  |  |  |  |  |  |  |  |  |  |  |  |  |  |  |  |  |  |  |  |  |  |  |
| 1. Boldness | 2.42 | .456 | 0.202* | -0.066 | -0.603** | -0.193* | -0.184* | 0.081 | -0.225* | -0.163* | 0.243** | (.83) |  |  |  |  |  |  |  |  |  |  |  |  |  |  |  |  |
| 1. Meanness | 1.72 | .417 | 0.142* | -0.290** | -0.221* | -0.555** | -0.408** | -0.306** | -0.397** | -0.251** | -0.225* | 0.231** | (.85) |  |  |  |  |  |  |  |  |  |  |  |  |  |  |  |
| 1. Disinhibition | 1.97 | .441 | 0.029 | -0.046 | 0.050 | -0.585** | -0.174* | -0.265** | -0.202* | -0.127* | -0.238** | -0.137* | 0.488** | (.83) |  |  |  |  |  |  |  |  |  |  |  |  |  |  |
| CAST |  |  |  |  |  |  |  |  |  |  |  |  |  |  |  |  |  |  |  |  |  |  |  |  |  |  |  |  |
| 1. Sadism (total) | 1.78 | .680 | -0.327** | 0.229** | 0.205* | 0.432** | 0.533** | 0.263** | 0.401** | 0.100 | 0.111 | -0.210* | -0.604** | -0.328** | (.87) |  |  |  |  |  |  |  |  |  |  |  |  |  |
| 1. Direct Verbal | 2.08 | .913 | -0.146* | 0.236** | 0.183* | 0.338** | 0.407** | 0.254** | 0.319** | 0.085 | 0.207* | -0.070 | -0.477** | -0.312** | 0.815** | (.78) |  |  |  |  |  |  |  |  |  |  |  |  |
| 1. Direct Physical | 1.38 | .686 | -0.158* | 0.106 | 0.147* | 0.347** | 0.244** | 0.186* | 0.335** | 0.029 | 0.059 | -0.180* | -0.505** | -0.239** | 0.777** | 0.525** | (.89) |  |  |  |  |  |  |  |  |  |  |  |
| 1. Vicarious | 1.90 | .957 | -0.446** | 0.187* | 0.158* | 0.351** | 0.574** | 0.185* | 0.310** | 0.111 | -0.003 | -0.251** | -0.470** | -0.231** | 0.798** | 0.407** | 0.439** | (.84) |  |  |  |  |  |  |  |  |  |  |
| ACME |  |  |  |  |  |  |  |  |  |  |  |  |  |  |  |  |  |  |  |  |  |  |  |  |  |  |  |  |
| 1. Affective Resonance | 4.21 | .576 | 0.117 | -0.080 | -0.060 | -0.355** | -0.242** | -0.240** | -0.327** | 0.083 | -0.056 | 0.065 | 0.653** | 0.275** | -0.497** | -0.409** | -0.472** | -0.332** | (.86) |  |  |  |  |  |  |  |  |  |
| 1. Cognitive Empathy | 3.72 | .616 | 0.153* | 0.201* | 0.260** | 0.030 | 0.005 | 0.017 | -0.078 | 0.323** | -0.005 | -0.145* | 0.138 | 0.068 | -0.218* | -0.118 | -0.198* | -0.210* | 0.436** | (.88) |  |  |  |  |  |  |  |  |
| 1. Affective Dissonance | 4.13 | .708 | 0.106 | -0.149* | -0.095 | -0.463** | -0.579** | -0.358** | -0.422** | -0.101 | -0.212 | 0.061 | 0.644** | 0.362** | -0.634** | -0.574** | -0.53** | -0.425** | 0.588** | 0.203* | (.90) |  |  |  |  |  |  |  |
| ASI-3 |  |  |  |  |  |  |  |  |  |  |  |  |  |  |  |  |  |  |  |  |  |  |  |  |  |  |  |  |
| 1. Cognitive Anxiety | 1.41 | 1.048 | 0.105 | 0.098 | -0.158* | 0.153* | 0.007 | 0.267** | -0.026 | 0.200* | 0.472** | 0.417** | -0.186* | -0.436** | 0.060 | 0.107 | 0.048 | -0.009 | -0.103 | -0.041 | -0.249* | (.88) |  |  |  |  |  |  |
| 1. Social Anxiety | 1.90 | .925 | 0.116 | 0.013 | -0.251** | -0.135 | -0.020 | 0.098 | -0.054 | 0.199* | 0.390** | 0.444** | 0.018 | -0.220* | -0.075 | 0.022 | -0.091 | -0.116 | 0.042 | -0.045 | -0.049 | 0.600** | (.78) |  |  |  |  |  |
| 1. Physical Anxiety | 1.49 | 1.051 | 0.158* | 0.065 | -0.145* | 0.131* | -0.021 | 0.267** | 0.054 | 0.204* | 0.381** | 0.399** | -0.149* | -0.387** | 0.033 | 0.114 | 0.067 | -0.086 | -0.095 | -0.035 | -0.258** | 0.744** | 0.550** | (.87) |  |  |  |  |
| BIS/BAS |  |  |  |  |  |  |  |  |  |  |  |  |  |  |  |  |  |  |  |  |  |  |  |  |  |  |  |  |
| 1. BAS Drive | 2.71 | .576 | 0.054 | -0.255** | -0.344** | -0.278** | -0.207* | -0.080 | -0.270** | -0.329** | -0.103 | 0.402** | 0.282** | 0.155* | -0.207* | -0.182* | -0.177* | -0.140 | -0.035 | -0.171* | 0.049 | 0.017 | 0.102 | 0.035 | (.72) |  |  |  |
| 1. BAS Fun Seeking | 3.04 | .547 | 0.011 | -0.088 | -0.229** | -0.203* | -0.127 | 0.038 | -0.230** | -0.165* | 0.005 | 0.360** | 0.102 | 0.179* | -0.034 | -0.067 | 0.107 | -0.086 | -0.133 | -0.163* | -0.058 | 0.047 | 0.128 | 0.062 | 0.480** | (.65) |  |  |
| 1. BAS Reward | 3.51 | .434 | -0.009 | -0.256** | -0.111 | 0.105 | -0.036 | 0.089 | -0.103 | -0.326** | -0.084 | 0.049 | -0.157* | -0.093 | 0.146* | 0.041 | 0.264** | 0.083 | -0.298** | -0.241** | -0.172* | -0.033 | -0.070 | -0.092 | 0.318** | 0.507** | (.70) |  |
| 1. BIS | 3.20 | .487 | -0.285** | 0.065 | 0.253** | 0.238** | 0.144* | 0.019 | 0.208* | -0.096 | -0.345** | -0.548** | -0.310** | -0.093 | 0.332** | 0.118 | 0.342** | 0.351** | -0.333** | -0.146* | -0.156* | -0.340** | -0.382** | -0.371** | -0.127 | -0.092 | 0.268** | (.74) |

*Note:* *n* = 191. SD4 = Short Dark Tetrad. TDMS = Two-Dimensional Machiavellianism Scale. B-PNI = Brief Pathological Narcissism Scale. TriPM = Triarchic Psychopathy Measure. CAST = Comprehensive Assessment of Sadistic Tendencies. ACME = Affective and Cognitive Measures of Empathy. ASI-3 = Anxiety Sensitivity Index. BIS/BAS = Behavioural Inhibition System/Behavioural Activation (Approach) System. Gender: man = 1, woman = 2 () = Cronbach’s alpha.

**p* < .05 ***p* < .001

**Table S5**

*Study 2 Multiple Regressions Predicting AACT Switch Point and Phase 2 Negative Approach from the Dark Tetrad (Model 1) and the Dark Tetrad and Gender (Model 2)*

| Model | Variable | *B* | *SE* | β | *t* | *p* |
| --- | --- | --- | --- | --- | --- | --- |
| Switch Point | | | | | | |
| 1 | SD4 Machiavellianism | -.191 | .140 | -.102 | -1.365 | .174 |
|  | SD4 Narcissism | .222 | .121 | .141 | 1.833 | .068 |
|  | SD4 Psychopathy | .141 | .136 | .085 | 1.031 | .304 |
|  | SD4 Sadism | -.232 | .109 | -.174 | -2.136 | .034 |
| 2 | SD4 Machiavellianism | -.173 | .143 | -.092 | -1.210 | .228 |
|  | SD4 Narcissism | .210 | .120 | .136 | 1.749 | .082 |
|  | SD4 Psychopathy | .137 | .135 | .084 | 1.012 | .313 |
|  | SD4 Sadism | -.181 | .111 | -.138 | -1.636 | .104 |
|  | Gender (1 = Man, 2 = Woman) | .347 | .212 | .123 | 1.635 | .104 |
| Phase 2 Negative Approach | | | | | | |
| 1 | SD4 Machiavellianism | .064 | .038 | .127 | 1.690 | .093 |
|  | SD4 Narcissism | -.052 | .033 | -.121 | -1.572 | .118 |
|  | SD4 Psychopathy | -.032 | .037 | -.070 | -0.852 | .395 |
|  | SD4 Sadism | .054 | .029 | .151 | 1.839 | .068 |
| 2 | SD4 Machiavellianism | .057 | .039 | .111 | 1.466 | .144 |
|  | SD4 Narcissism | -.049 | .033 | -.117 | -1.505 | .134 |
|  | SD4 Psychopathy | -.028 | .037 | -.062 | -0.754 | .452 |
|  | SD4 Sadism | .042 | .030 | .118 | 1.391 | .166 |
|  | Gender (1 = Man, 2 = Woman) | -.106 | .058 | -.138 | -1.833 | .068 |

*Note*. Valid *n*’s = 185 – 191.

**Table S6**

*Study 3 Intercorrelations*

| Scale | *M* | *SD* | Gender | 1 | 2 | 3 | 4 | 5 | 6 | 7 |
| --- | --- | --- | --- | --- | --- | --- | --- | --- | --- | --- |
| SD4 |  |  |  |  |  |  |  |  |  |  |
| 1. Machiavellianism | 3.32 | .616 | -0.185* | (.71) |  |  |  |  |  |  |
| 1. Narcissism | 2.50 | .755 | -0.166* | 0.328** | (.81) |  |  |  |  |  |
| 1. Psychopathy | 1.83 | .704 | -0.257** | 0.328** | 0.446** | (.82) |  |  |  |  |
| 1. Sadism | 2.24 | .870 | -0.495** | 0.407** | 0.242** | 0.555** | (.84) |  |  |  |
| ACME |  |  |  |  |  |  |  |  |  |  |
| 1. Affective Resonance | 4.21 | .614 | 0.240** | -0.239** | -0.045 | -0.384** | -0.444** | (.61) |  |  |
| 1. Cognitive Empathy | 3.72 | .615 | 0.134* | -0.110 | 0.226** | -0.106 | -0.123* | 0.396** | (.91) |  |
| 1. Affective Dissonance | 4.43 | .593 | 0.338** | -0.351** | -0.294** | -0.587** | -0.660** | 0.634** | 0.206** | (.90) |

*Note:* *n* = 288. SD4 = Short Dark Tetrad. ACME = Affective and Cognitive Measures of Empathy. Gender: man = 1, woman = 2. () = Cronbach’s alpha.

**p* < .05 ***p* < .001

**Table S7**

*Study 3 Multiple Regressions Predicting AACT Switch Point and Phase 2 Negative Approach from the Dark Tetrad (Model 1) and the Dark Tetrad and Gender (Model 2)*

| Model | Variable | *B* | *SE* | β | *t* | *p* |
| --- | --- | --- | --- | --- | --- | --- |
| Switch Point | | | | | | |
| 1 | SD4 Machiavellianism | -.075 | .114 | -.044 | -0.660 | .510 |
|  | SD4 Narcissism | -.122 | .094 | -.087 | -1.292 | .197 |
|  | SD4 Psychopathy | .025 | .115 | .016 | 0.215 | .830 |
|  | SD4 Sadism | -.135 | .090 | -.111 | -1.505 | .133 |
| 2 | SD4 Machiavellianism | -.082 | .115 | -.048 | -0.713 | .477 |
|  | SD4 Narcissism | -.109 | .095 | -.078 | -1.153 | .250 |
|  | SD4 Psychopathy | .016 | .115 | .010 | 0.137 | .891 |
|  | SD4 Sadism | -.096 | .100 | -.079 | -0.967 | .334 |
|  | Gender (1 = Man, 2 = Woman) | .124 | .144 | .059 | 0.861 | .390 |
| Phase 2 Negative Approach | | | | | | |
| 1 | SD4 Machiavellianism | .024 | .031 | .051 | 0.774 | .439 |
|  | SD4 Narcissism | .032 | .026 | .084 | 1.245 | .214 |
|  | SD4 Psychopathy | -.010 | .031 | -.025 | -0.328 | .743 |
|  | SD4 Sadism | .039 | .024 | .117 | 1.588 | .113 |
| 2 | SD4 Machiavellianism | .024 | .031 | .051 | 0.758 | .449 |
|  | SD4 Narcissism | .030 | .026 | .079 | 1.166 | .245 |
|  | SD4 Psychopathy | -.008 | .031 | -.020 | -0.256 | .798 |
|  | SD4 Sadism | .035 | .027 | .105 | 1.277 | .203 |
|  | Gender (1 = Man, 2 = Woman) | -.014 | .039 | -.024 | -0.357 | .721 |

*Note*. Valid *n*’s = 286 – 288.

**Table S8**

*Study 4 Intercorrelations*

| Scale | *M* | *SD* | Gender | 1 | 2 | 3 | 4 | 5 | 6 | 7 |
| --- | --- | --- | --- | --- | --- | --- | --- | --- | --- | --- |
| SD4 |  |  |  |  |  |  |  |  |  |  |
| 1. Machiavellianism | 3.33 | .573 | -0.211** | (.71) |  |  |  |  |  |  |
| 1. Narcissism | 2.45 | .737 | -0.261** | 0.358** | (.83) |  |  |  |  |  |
| 1. Psychopathy | 1.85 | .692 | -0.367** | 0.251** | 0.409** | (.81) |  |  |  |  |
| 1. Sadism | 2.21 | .865 | -0.515** | 0.426** | 0.271** | 0.568** | (.85) |  |  |  |
| ACME |  |  |  |  |  |  |  |  |  |  |
| 1. Affective Resonance | 4.16 | .574 | 0.333** | -0.274** | -0.110 | -0.487** | -0.487** | (.86) |  |  |
| 1. Cognitive Empathy | 3.65 | .618 | 0.140* | 0.008 | 0.138* | -0.193** | -0.206** | 0.368** | (.91) |  |
| 1. Affective Dissonance | 4.36 | .594 | 0.364** | -0.361** | -0.292** | -0.546** | -0.623** | 0.721** | 0.223** | (.90) |

*Note:* *n* = 276. SD4 = Short Dark Tetrad. ACME = Affective and Cognitive Measures of Empathy. Gender: man = 1, woman = 2. () = Cronbach’s alpha.

**p* < .05 ***p* < .001.

**Table S9**

*Study 4 Multiple Regressions Predicting AACT Switch Point and Phase 2 Negative Approach from the Dark Tetrad (Model 1) and the Dark Tetrad and Gender (Model 2)*

| Model | Variable | *B* | *SE* | β | *t* | *p* |
| --- | --- | --- | --- | --- | --- | --- |
| Switch Point | | | | | | |
| 1 | SD4 Machiavellianism | -.120 | .123 | -.067 | -0.973 | .331 |
|  | SD4 Narcissism | -.193 | .095 | -.138 | -2.031 | .043 |
|  | SD4 Psychopathy | -.038 | .114 | -.025 | -0.334 | .739 |
|  | SD4 Sadism | -.078 | .092 | -.066 | -0.854 | .394 |
| 2 | SD4 Machiavellianism | -.125 | .123 | -.070 | -1.019 | .309 |
|  | SD4 Narcissism | -.186 | .096 | -.134 | -1.932 | .054 |
|  | SD4 Psychopathy | -.048 | .114 | -.033 | -0.425 | .671 |
|  | SD4 Sadism | -.041 | .099 | -.035 | -0.416 | .678 |
|  | Gender (1 = Man, 2 = Woman) | .101 | .144 | .049 | 0.702 | .484 |
| Phase 2 Negative Approach | | | | | | |
| 1 | SD4 Machiavellianism | .054 | .034 | .107 | 1.577 | .116 |
|  | SD4 Narcissism | .064 | .027 | .163 | 2.419 | .016 |
|  | SD4 Psychopathy | .008 | .032 | .018 | 0.244 | .808 |
|  | SD4 Sadism | .020 | .026 | .058 | 0.759 | .449 |
| 2 | SD4 Machiavellianism | .056 | .034 | .110 | 1.614 | .108 |
|  | SD4 Narcissism | .062 | .027 | .158 | 2.304 | .022 |
|  | SD4 Psychopathy | .010 | .032 | .023 | 0.306 | .760 |
|  | SD4 Sadism | .007 | .028 | .019 | 0.234 | .815 |
|  | Gender (1 = Man, 2 = Woman) | -.034 | .040 | -.059 | -0.853 | .394 |

*Note*. Valid *n*’s = 271 – 276.

**Table S10**

*Study 3 Multiple Regressions Predicting VTT Behaviour from the Dark Tetrad*

| Variable | *B* | *SE* | β | *t* | *p* |
| --- | --- | --- | --- | --- | --- |
| Positive Viewing Time | | | | | |
| SD4 Machiavellianism | -.123 | .186 | -.049 | -0.660 | .510 |
| SD4 Narcissism | -.016 | .157 | -.008 | -0.103 | .918 |
| SD4 Psychopathy | -.055 | .193 | -.025 | -0.285 | .776 |
| SD4 Sadism | -.378 | .148 | -.213 | -2.550 | .011 |
| Negative Viewing Time | | | | | |
| SD4 Machiavellianism | .626 | .375 | .127 | 1.670 | .096 |
| SD4 Narcissism | -.294 | .316 | -.072 | -0.928 | .355 |
| SD4 Psychopathy | .438 | .388 | .100 | 1.127 | .261 |
| SD4 Sadism | -.187 | .298 | -.054 | -0.629 | .530 |
| Positive Up Presses | | | | | |
| SD4 Machiavellianism | -2.825 | 1.238 | -.170 | -2.283 | .023 |
| SD4 Narcissism | 1.087 | 1.045 | .079 | 1.039 | .300 |
| SD4 Psychopathy | -1.616 | 1.283 | -.110 | -1.259 | .209 |
| SD4 Sadism | -.627 | .984 | -.053 | -0.637 | .525 |
| Negative Up Presses | | | | | |
| SD4 Machiavellianism | 1.453 | 1.739 | .063 | 0.835 | .404 |
| SD4 Narcissism | .666 | 1.469 | .035 | 0.453 | .651 |
| SD4 Psychopathy | 1.876 | 1.803 | .093 | 1.040 | .299 |
| SD4 Sadism | .051 | 1.383 | .003 | 0.037 | .971 |
| Positive Down Presses | | | | | |
| SD4 Machiavellianism | .054 | .454 | .009 | 0.119 | .905 |
| SD4 Narcissism | .660 | .384 | .132 | 1.721 | .087 |
| SD4 Psychopathy | -.105 | .471 | -.020 | -0.222 | .824 |
| SD4 Sadism | .730 | .361 | .170 | 2.022 | .044 |
| Negative Down Presses | | | | | |
| SD4 Machiavellianism | -.453 | .516 | -.066 | -0.877 | .382 |
| SD4 Narcissism | 1.247 | .436 | .220 | 2.858 | .005 |
| SD4 Psychopathy | -.526 | .535 | -.087 | -0.983 | .327 |
| SD4 Sadism | .328 | .411 | .067 | 0.799 | .425 |

*Note*. Valid *n* = 207.

Table S11

*Study 4 Multiple Regressions Predicting VTT Behaviour from the Dark Tetrad*

| Variable | *B* | *SE* | β | *t* | *p* |
| --- | --- | --- | --- | --- | --- |
| Positive Viewing Time | | | | | |
| SD4 Machiavellianism | .027 | .314 | .007 | 0.087 | .931 |
| SD4 Narcissism | -.053 | .246 | -.016 | -0.214 | .830 |
| SD4 Psychopathy | .291 | .288 | .086 | 1.010 | .314 |
| SD4 Sadism | -.420 | .237 | -.155 | -1.773 | .078 |
| Negative Viewing Time | | | | | |
| SD4 Machiavellianism | .462 | .152 | .229 | 3.039 | .003 |
| SD4 Narcissism | -.069 | .119 | -.044 | -0.583 | .561 |
| SD4 Psychopathy | .271 | .139 | .162 | 1.944 | .053 |
| SD4 Sadism | -.280 | .115 | -.210 | -2.446 | .015 |
| Positive Up Presses | | | | | |
| SD4 Machiavellianism | .214 | 1.708 | .010 | 0.125 | .900 |
| SD4 Narcissism | 2.437 | 1.337 | .138 | 1.822 | .070 |
| SD4 Psychopathy | .632 | 1.566 | .034 | 0.404 | .687 |
| SD4 Sadism | -1.930 | 1.287 | -.130 | -1.500 | .135 |
| Negative Up Presses | | | | | |
| SD4 Machiavellianism | 1.621 | .749 | .164 | 2.163 | .032 |
| SD4 Narcissism | .300 | .587 | .038 | 0.511 | .610 |
| SD4 Psychopathy | 1.112 | .687 | .136 | 1.619 | .107 |
| SD4 Sadism | -1.306 | .565 | -.199 | -2.312 | .022 |
| Positive Down Presses | | | | | |
| SD4 Machiavellianism | -.090 | .528 | -.013 | -0.171 | .864 |
| SD4 Narcissism | .327 | .413 | .058 | 0.792 | .429 |
| SD4 Psychopathy | -.209 | .484 | -.035 | -0.431 | .667 |
| SD4 Sadism | 1.483 | .398 | .312 | 3.727 | <.001 |
| Negative Down Presses | | | | | |
| SD4 Machiavellianism | -.978 | .517 | -.145 | -1.892 | .060 |
| SD4 Narcissism | .216 | .405 | .041 | 0.533 | .595 |
| SD4 Psychopathy | -.303 | .474 | -.055 | -0.641 | .522 |
| SD4 Sadism | .593 | .389 | .133 | 1.522 | .129 |

*Note*. Valid *n* = 230.
